# Unravelling the synthetic and therapeutic aspects of five, six and fused heterocycles using Vilsmeier–Haack reagent

**DOI:** 10.1039/d3ra04309f

**Published:** 2023-09-05

**Authors:** Mamta Chahal, Sudeep Dhillon, Priyanka Rani, Ginna Kumari, Deepak Kumar Aneja, Mayank Kinger

**Affiliations:** a Department of Chemistry, Chaudhary Bansi Lal University Bhiwani 127021 Haryana India drmayankkinger@cblu.ac.in

## Abstract

The aim of this review is to encapsulate the synthetic protocols and medicinal aspects of a wide range of heterocyclic compounds using the Vilsmeier–Haack (V. H.) reagent. These derivatives act as excellent precursors having different aryl ring functionalities and could be used for the synthesis of a variety of heterocyclic scaffolds. The V. H. reagent, a versatile reagent in organic chemistry, is used to formylate various heterocyclic compounds of medicinal interest. Due to the different chemical interactions, efficacy, and potency of V. H. reagents, plenty of heterocyclic compounds can be synthesized which serve as a constituent in various novel medications and acts as a bridge between biology and chemistry. These carboxylate moieties can effectively cooperate as precursors for several multi-component reactions (MCR) including Strecker synthesis, Bucherer–Berg reaction and post-MCR cyclization, modified variants with various pharmaceutical applications such as anti-tumor, anti-convulsant, anti-chitosomal and so on.

## Abstract 2

The aim of this review is to encapsulate the synthetic protocols and medicinal aspects of a wide range of heterocyclic compounds using the Vilsmeier–Haack (V. H.) reagent.

## Graphical abstract

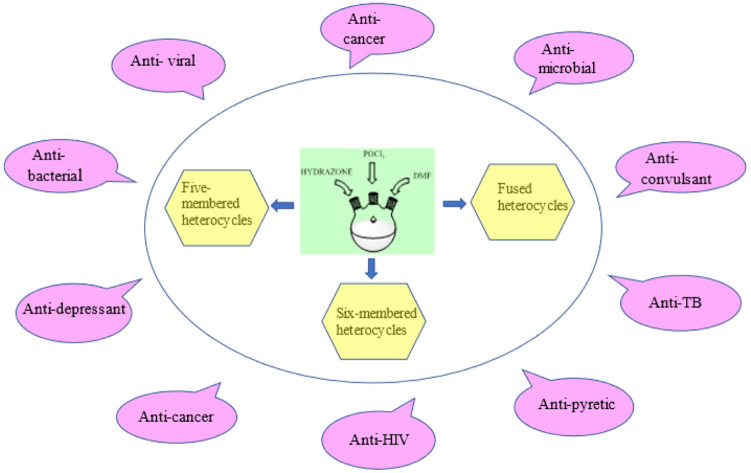

## 1. Introduction

**Fig. 1:**
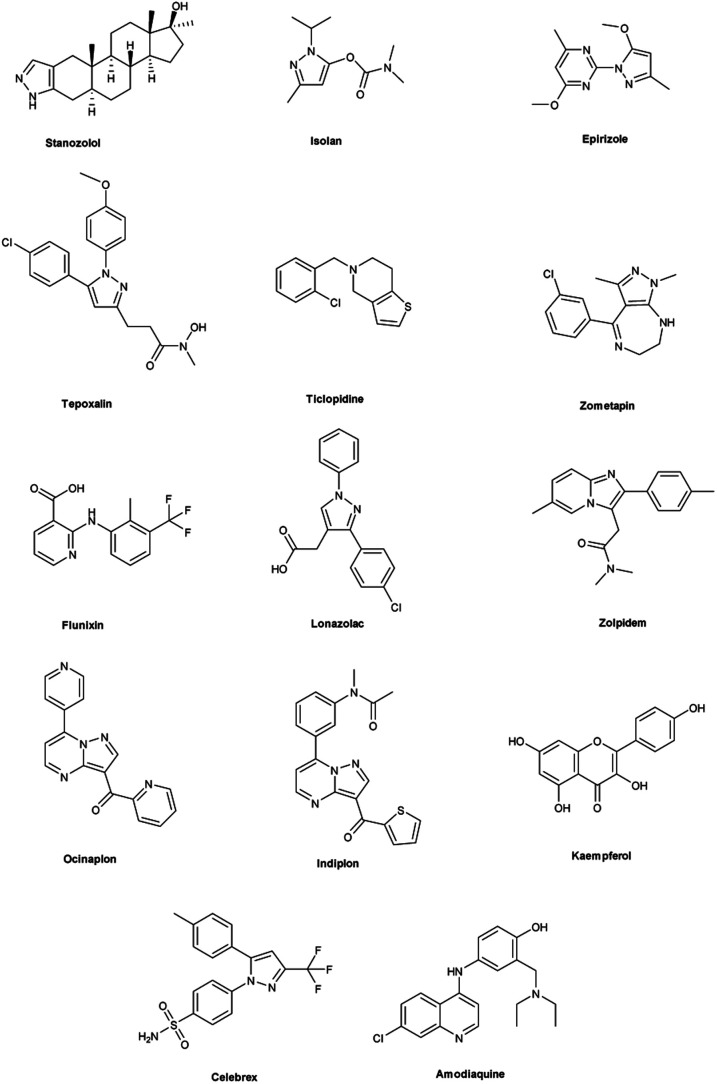
Commercial drugs and bioactive molecules containing heterocyclic moieties.

Vilsmeier–Haack (V. H.) reaction^1,2^ is named after Anton Vilsmeier and Albrecht Haack in 1927, which is an efficient, affordable and mild reagent used to transform an electron rich aromatic ring to an aryl aldehyde using dimethylformamide (DMF) and phosphorus oxychloride (POCl_3_).^3^ The compounds that include at least one heteroatom *viz.* nitrogen, oxygen, sulphur *etc.* as a member of the ring system are known as heterocycles.^4,5^ The implication of heterocyclic scaffolds is reflected in the field of medicinal chemistry as an interesting template for the design, synthesis and development of biologically active molecules or drugs including DNA, RNA, chlorophyll, haemoglobin, vitamins and others.^6,7^ Numerous examples of drugs and bioactive molecules containing different heterocyclic core in their molecular architecture can be found in the literature and also used as agrochemicals in the form of herbicides, fungicides and insecticides to protect crops.^8^ Literature review reveals that more than 85% of all physiologically active chemical compounds includes heterocycles, thereby emphasizes their significant role in the modern drugs design.^9^ In addition, many drugs have been approved and successfully marketed by the FDA in recent years, such as insecticide fipronil,^10^ azo dye tartrazine^11^ used as food colouring, sildenafil^12^ used to treat erectile dysfunction, dipyrone^13^ a strong analgesic and anti-pyretic, celecoxib^14^ used to relieve pain, zometapine^15^ used to reduces depression, celebrex^16^ and ionazolac^17^ used against inflammation, rimonabant^18^ used to reduce obesity *etc.* (Fig. 1).

Vilsmeier–Haack (V. H.) reagent is an important structural unit in heterocyclic chemistry and occupy a prominent position in the field of medicinal chemistry due to its remarkable pharmacological activities such as anti-fungal,^19^ anti-angiogenesis,^20^ anti-cancer,^21^ anti-inflammatory,^22^ anti-depressant,^23^ anti-tubercular,^24^ anti-viral,^25^ anti-convulsant,^26^ anti-pyretic,^27^ anti-tumor,^28^ anti-HIV,^29,30^ anti-TB,^31^ anti-proliferative,^32^ anti-analgesic,^33^ anti-fertility^34^ and anti-bacterial^35^*etc.*

**Fig. 2:**
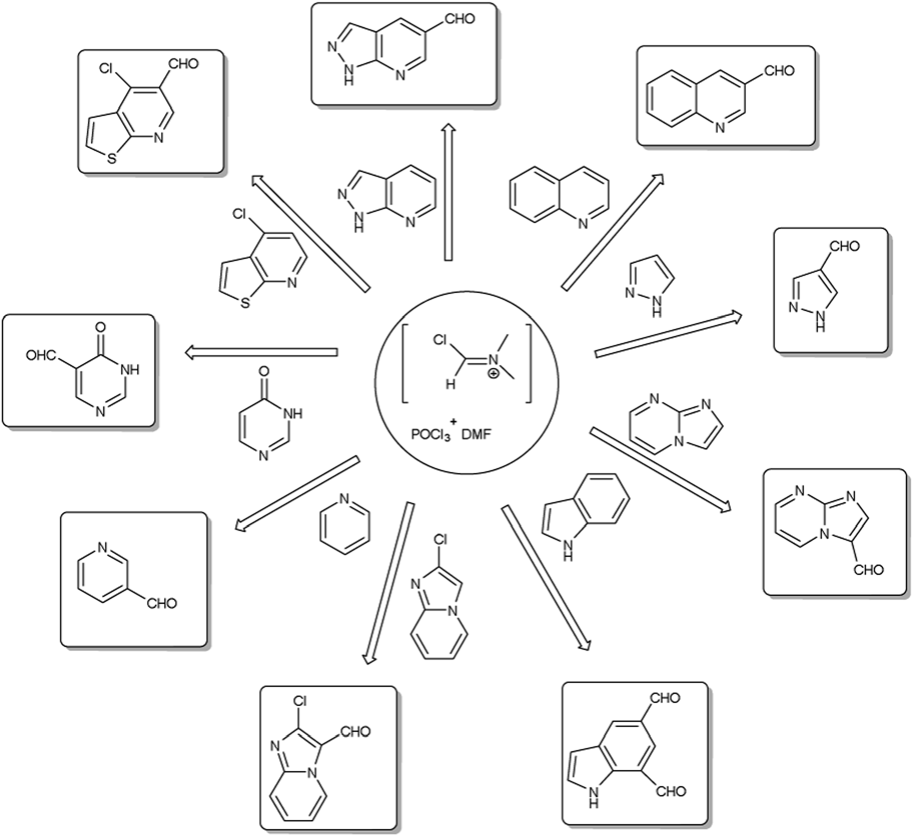
Synthesis of several heterocyclic precursors using V. H. reagent.

A pictorial representation of several heterocyclic framework synthesized using V. H. reagent is given in Fig. 2.

Keeping in view of the above, we recapitulated the recent progress in the development of enumerate synthetic routes of heterocyclic derivatives using different substrates with the help of V. H. reagent. This review is divided into three parts *viz.* synthesis and biological perspective of five-membered, six-membered and fused rings heterocycles. First section comprising synthesis of five-membered heterocyclic compounds which has been sub-divided into three categories *i.e.*, cyclization followed by formylation, formylation, miscellaneous while second section includes synthesis of six-membered heterocyclic compounds has been categorized into formylation of pyridine carbaldehyde and miscellaneous. The last section of this segment contains formylation of numerous fused heterocyclic ring system *i.e.*, quinoline, imidazo-pyrimidine, imidazo-pyridine *etc.*

## 2. Synthesis of five-membered heterocyclic compounds

### 2.1 Cyclization and formylation of pyrazole

**Fig. 3:**
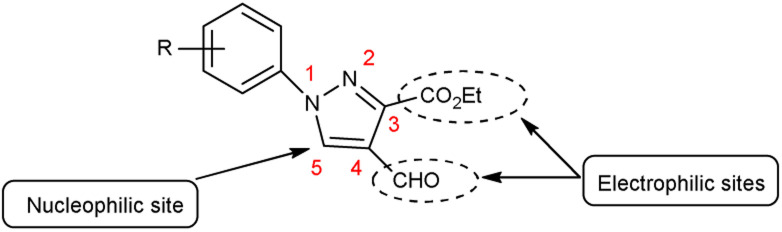
Pyrazole.

Pyrazoles,^36^ also known as azoles, are five-membered heterocyclic compounds with two closely spaced nitrogen atoms. The chemical reactivity of the pyrazole molecule can be explained by the influence of the individual atoms. The *N* atom in position 2 reacts with electrophiles because it is more basic and has two electrons. The *N* atom in position 1 is not reactive, but loses its proton in the presence of a base. The combined action of the two *N* atoms causes a reduction in the charge density on C-3 and C-5, exposing C-4 to electrophilic attack (Fig. 3).

**Fig. 4:**
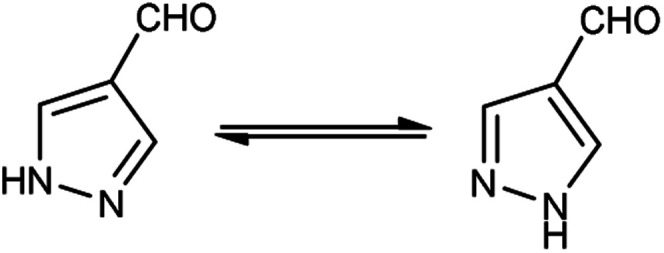
Tautomer's of pyrazole carbaldehyde.

**Fig. 5:**
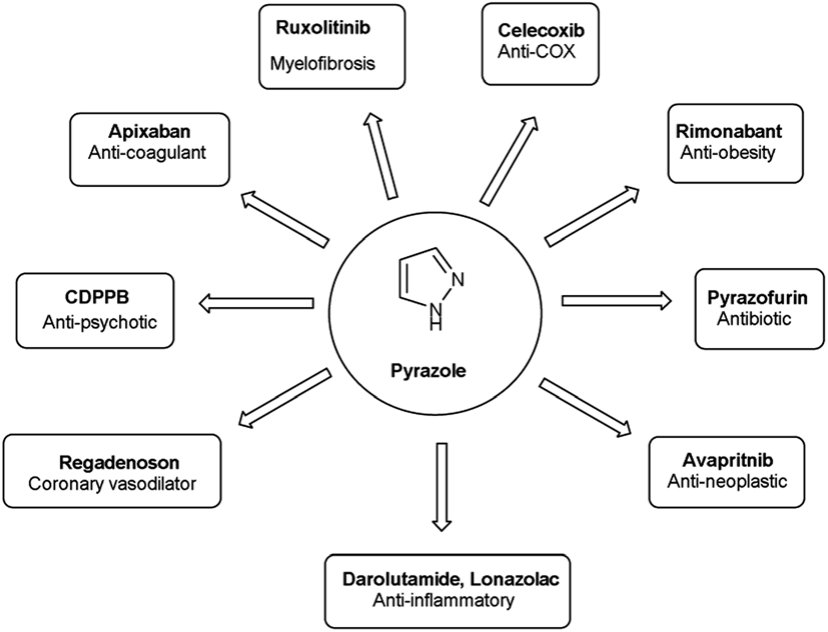
Some pyrazole-based drugs available in the market.

Different tautomeric forms of pyrazole carbaldehyde are outlined in Fig. 4. The pyrazole nucleus has been known to exhibited a wide range of biological activities including insecticidal, fungicidal, anti-bacterial, anti-viral, anti-tumor, anti-histaminic and anti-depressant agent have been shown in Fig. 5.

**Scheme 1:**
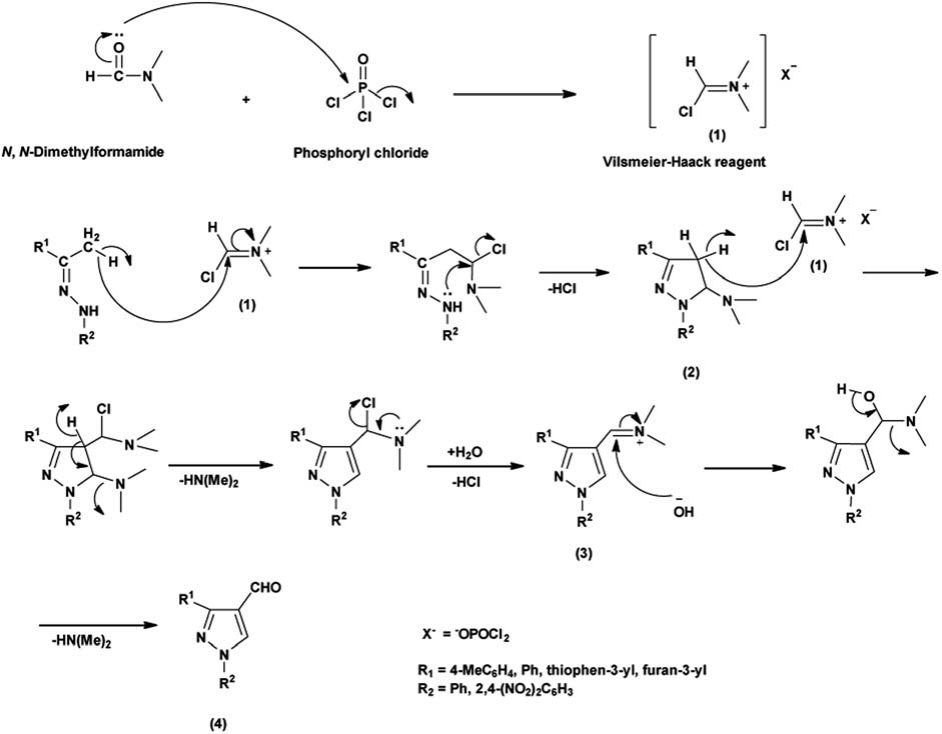
Plausible mechanism for formylation of pyrazole ring.

An elegant synthetic route for the formylation of pyrazoles involves the generation of V. H. reagent *i.e.*, iminium salt (1) in the first step. In the next step, electron-rich carbon encounters the iminium salt (1) leading to deprotonation followed by cyclization and exclusion of chloride ion to generate intermediate (2). Further, the intermediate (2) another attack on iminium salt (1) culminating in the formation of intermediate (3). The hydrolysis of the intermediate (3) completes the transformation, resulting in the formation of formyl pyrazole (4)^37^ (Scheme 1).

**Scheme 2:**
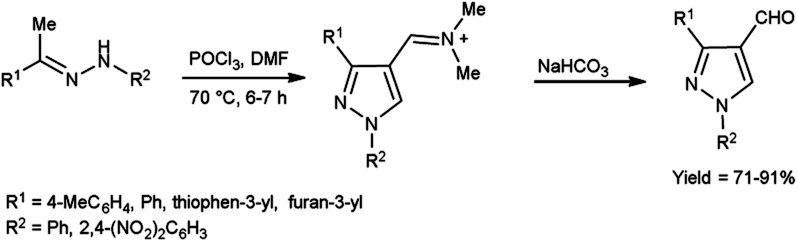
Synthesis of pyrazole carbaldehydes.

In 2022, El-Gharably *et al.*^37^ synthesized some pyrazole carbaldehydes on treatment of hydrazones with V. H. reagent (DMF-POCl_3_) in ice-bath for 1 hour and stirred up to 70 °C for 6 to 7 hours, in excellent yield. These formyl pyrazole derivatives were used to prepare Schiff’s bases further and screened for their anti-tumor, anti-fungal activities against *Aspergillus niger* (*A. niger*) as well as anti-bacterial activities against bacteria *Staphylococcus aureus* (*S. aureus*), *Bacillus cereus* (*B. cereus*) and *Escherichia coli* (*E. coli*). The results of the studies demonstrated that some of pyrazole-based Schiff’s bases (1-(2,4-dinitrophenyl)-3-phenyl-1*H*-pyrazole-4-carbaldehyde) exhibited more anti-microbial potential against *B. cereus* bacteria showed inhibition zone of 7.5 ± 0.6 mm and 1-phenyl-3-(furan-3-yl)-1*H*-pyrazole-4-carbaldehyde against bacteria *S. aureus* gave inhibition zone at 25 ± 2.0 mm. All synthesized products showed no activity against *E. coli* (Scheme 2).

**Scheme 3:**
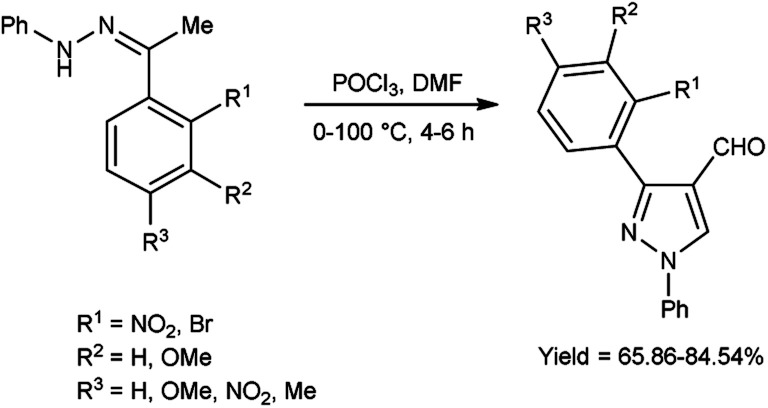
Synthesis of pyrazole carbaldehydes.

In 2020, Mishra and co-workers^38^ synthesized few pyrazole carbaldehyde from substituted hydrazone using V. H. reagent with stirring at 0–5 °C for 15 minutes followed by heating at 100 °C for 4 to 6 hours, in good to excellent yield. Further, different Schiff’s bases were produced using formyl pyrazole and different amines. These derivatives were evaluated for their *in vivo* anti-inflammatory activity using carrageenan-induced rat paw edema method and approved remarkable reduction of inflammation. Out of synthesized compounds, (18*E*)-*N*-(((3-(2-aminophenyl)-4,5-dihydro-1-phenyl-1*H*-pyrazole-3-yl)methylene-3-yl)methylene)-3-chlorobenzenamine and (18*E*)-2-bromo-*N*-((4,5-dihydro-3-(2-nitrophenyl)-1-phenyl-1*H*-pyrazole-4-yl)methylene)benzenamine were found more promising against infection when compared to diclofenac sodium as a reference drug (Scheme 3).

**Scheme 4:**
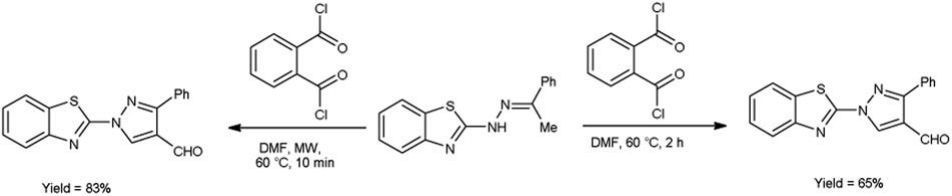
Synthesis of 4-formyl pyrazole.

In 2020, Kumari *et al.*^39^ reported the comparative study of conventional and microwave-assisted (MW) method for synthesis of 4-formyl pyrazole. In conventional method, phthaloyl dichloride (OPC)-DMF and substrate were stirred at 60 °C for 2 hours while in non-conventional method, phthaloyl dichloride (OPC)-DMF and substrate were heated in microwave at 60 °C for 10 minutes. In MW condition, cyclization of compound (*E*)-2-(2-(1-phenylethylidene)hydrazinyl)benzo[*d*]thiazole afforded the enhancement of reaction rate and yielded the compound 1-(benzo[*d*]thiazol-2-yl)-3-phenyl-1*H*-pyrazole-4-carbaldehyde from 65% to 83% yield in comparison to thermal condition. The efficient reaction conditions were optimized by performing a series of experiments under varying solvents which revealed in excellent yield of the expected product in 10 minutes when carried out in DMF. Advantage of this protocol in owing to its simplified operations, convenient process flow, high yield production and the ability to recycle the by-product. The reaction eliminated the need for separate reactions, streamlining the overall process. Due to which, it reduces the complexity of the synthesis, making it easier to perform and manage in the laboratory or on industrial scale contribute to a more efficient and environmentally conscious chemical synthesis. These aspects are crucial in modern organic synthesis, where researchers aim to develop greener and efficient methodologies for drug discovery, agrochemicals and industrial applications (Scheme 4).

**Scheme 5:**
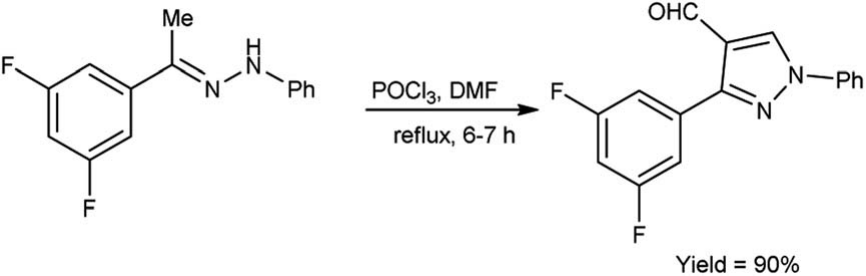
Synthesis of pyrazole carbaldehydes.

In 2019, Kumar and group^40^ developed a convenient synthesis of 3-(2,5-difluorophenyl)-1-phenyl-1*H*-pyrazole-4-carbaldehyde from (*E*)-1-[1-(3,5-difluorophenyl)ethylidene]-2-phenylhydrazine using V. H. reagent (DMF-POCl_3_) with stirring at 0 °C for 30 minutes followed by refluxing for 6 hours, in excellent yield. The earlier method reported was resulted in a 60% yield of the desired compound *i.e.*, formyl pyrazole on adding 2 equivalents of phosphorus oxychloride. However, the current protocol mentioned in scheme below has improved the yield from 60% to 90% by adding 10 equivalents of POCl_3_ (Scheme 5).

**Scheme 6:**
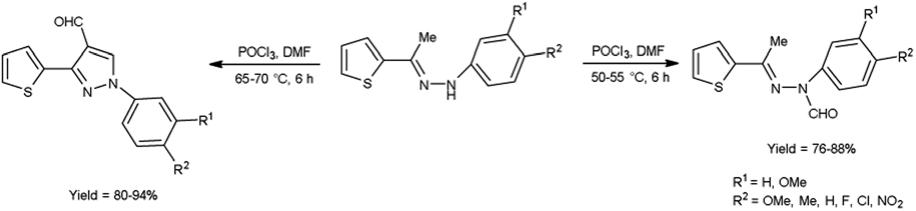
Synthesis of (*E*)-*N*-(aryl)-*N*-(1-thiophen-2-yl)ethylidene)formylhydrazide and 3-(thiophen-2-yl)-1*H*-pyrazole-4-carbaldehydes.

In 2015, Raghavendra *et al.*^41^ synthesized some (*E*)-*N*-(aryl)-3-(thiophen-2-yl)-1*H*-pyrazole-4-carbaldehyde when (*E*)-1-aryl-2-[(1-thiophen-2-yl)ethylidene]hydrazine using with V. H. reagent in cold condition followed by stirring at 55 °C for 6 hours, in excellent yield. The compound (*E*)-*N*-(aryl)-*N*′-(1-(thiophen-2-yl)ethylidene)formohydrazide was prepared when V. H. reagent reacted with (*E*)-1-aryl-2-[(1-thiophen-2-yl)ethylidene]hydrazine in ice-bath followed by stirring the reaction mixture at 75 °C for 6 hours, in excellent yield. According to this protocol, direct formylation appeared at nitrogen atom of substrate at 50 to 55 °C but cyclization and formylation occurred by using phosphorus oxychloride in excess and enhancing the temperature up to 75 °C. Paper disc diffusion method was employed for the anti-microbial activity of resulting compounds by using *E. coli*, *Salmonella typhimurium* (*S. typhimurium*), *B. subtilis*, *A. niger*, *Aspergillus flavus* (*A. flavus*) and *Fusarium oxysporum* strains. Among the synthesized compounds, (*E*)-*N*-(4-chlorophenyl)-*N*′-(1-(thiophen-2-yl)ethylidene)formohydrazide and 1-(4-chlorophenyl)-3-(thiophen-2-yl)-1*H*-pyrazole-4-carbaldehyde demonstrated good anti-bacterial activity when correlate with the reference drug streptomycin. The compounds with halogen substituted final products were found more active as compared to other functional groups *i.e.* methyl, methoxy, nitro *etc.* (Scheme 6).

**Scheme 7:**
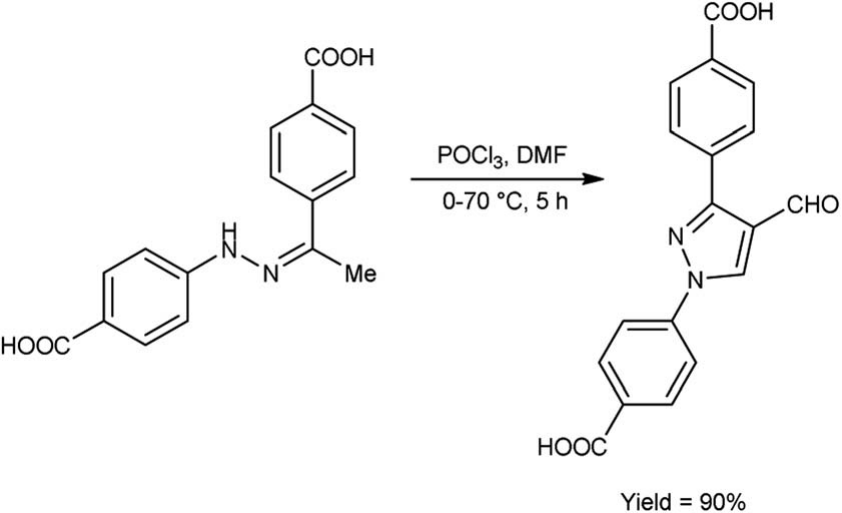
Synthesis of derivatives of 4,4-(4-formyl-1*H*-pyrazole-1,3-diyl)dibenzoic acid.

In 2020, Delancey and group^42^ produced 4,4-(4-formyl-1*H*-pyrazole-1,3-diyl)dibenzoic acid from the reaction of hydrazones with V. H. reagent at 0 °C followed by stirring the reaction mixture at 70 °C for 5 hours, in excellent yield. Further novel 2-(1-benzyl-3-(4-fluorophenyl)-1*H*-pyrazol-4-yl)-7-fluoro-4*H*-chromen-4-ones were synthesized by utilizing this formyl pyrazole as substrate. The potential toxicity of synthetic compounds was evaluated using human NCI-60 cancer cell and embryonic kidney (HEK293) cell lines. At 10 M concentration, these compounds did not suppress the progress of the NCI-60 cancer cell lines. The *N*,*N*-disubstituted compounds did not show any activity when screened against these strains. Structure–activity relationship (SAR) studies proved that halogen-substituted compounds showed higher potency against anti-bacterial infection. The chloro-substituted product, 4,4′-(4-{(*E*)-[2-(4-chlorophenyl)hydrazinylidene]methyl}-1*H*-pyrazole-1,3-diyl)dibenzoic acid exhibited good potency against *Acinetobacter baumannii* of different strains, ATCC 747 with MIC value of 1.56 μg mL^−1^ and ATCC BAA-1605 with MIC value of 3.125 μg mL^1^ respectively. The bromo-substituted product, 4,4′-(4-{(*E*)-[2-(3-bromophenyl)hydrazinylidene]methyl}-1*H*-pyrazole-1,3-diyl)dibenzoic acid showed excellent potency with MIC value of 3.125 μg mL^−1^. The biological activity of the resultant products was reduced with increasing the number of halogen atoms since penta-substituted compounds showed no activity (Scheme 7).

**Scheme 8:**
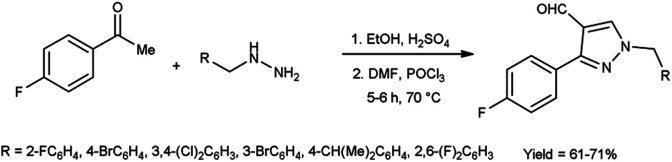
Synthesis of 1-substitutedbenzyl-3-(4-fluorophenyl)-1*H*-pyrazole-4-carbaldehydes.

In 2020, Hon *et al.*^43^ described the cyclization and formylation of 1-benzyl-2-(1-(4-fluorophenyl)ethylidene)hydrazones to synthesize 1-benzyl-3-(4-fluorophenyl)-1*H*-pyrazole-4-carbaldehydes using V. H. reagent (DMF-POCl_3_) in cold condition and stirred further at 70 °C for 5 to 6 hours, in good yield. The synthesized compounds were screened against anti-bacterial efficacy. The well diffusion method at 1 mg mL^−1^ concentration was used to analyse the bacterial strains *S. aureus*, *B. subtilis*, *E. coli* and *P. aeruginosa*. The compound, 1-(4-bromo-2-fluorobenzyl)-3-(4-fluorophenyl)-1*H*-pyrazole-4-carbaldehyde showed good activity against *E. coli* and *P. aeruginosa* strains. The compound 1-(4-isopropylbenzyl)-3-(4-fluorophenyl)-1*H*-pyrazole-4-carbaldehyde was found more potent against *P. aeruginosa, B. subtilis* and *S. aureus etc.* when compared with the standard drug ampicillin (Scheme 8).

**Scheme 9:**
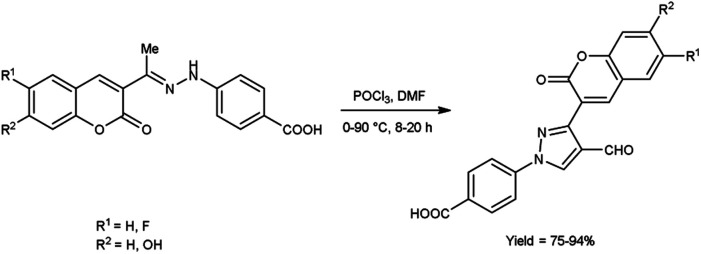
Synthesis of substituted-formyls pyrazole derivatives.

In 2020, Alnufaie and co-workers^44^ described the preparation of coumarin-substituted formyl pyrazole from different hydrazones using V. H. reagent (DMF-POCl_3_) in ice-bath for 30 minutes followed by stirring the reaction mixture at 90 °C for 8 to 20 hours, in good to excellent yield. The reaction mixture was poured into the crushed ice and stirred for 12 hours. Further, using these formyl pyrazoles, some coumarin-based hydrazones were produced. These synthesized compounds were screened against seven Gram-positive strains and three Gram-negative bacterial strains using time kill assay, biofilm inhibition assay and biofilm destruction assay. Amongst the synthesized compounds, 4-[4-[(*E*)-(diphenylhydrazono)methyl]-3-(6-fluoro-2-oxo-3,8*a*-dihydrochromen-3-yl)pyrazol-1-yl]benzoic acid showed resistant against three different strains of *S. aureus* ATCC 33592 (Sa92), ATCC 700699 (Sa99) and ATCC 33591 (Sa91), with both MIC value as low as 1.56 μg mL^−1^ and 3.125 μg mL^−1^. The results of the study demonstrated that fluoro-substituted compound, 4-[3-(7-fluoro-2-oxo-3,8*a*-dihydrochromen-3-yl)-4-[(*E*)-[[4-(trifluoromethyl)phenyl]hydrazono]methyl]pyrazol-1-yl]benzoic acid was found most active while chloro and bromo-substituted compounds were found moderately active. On the other hand, no activity was reported when hydroxy-substituted compounds were screened. The resultant compounds exhibited very little toxicity when compared the IC_50_ value of HEK293 cells to the MIC value of bacterial strains (Scheme 9).

**Scheme 10:**
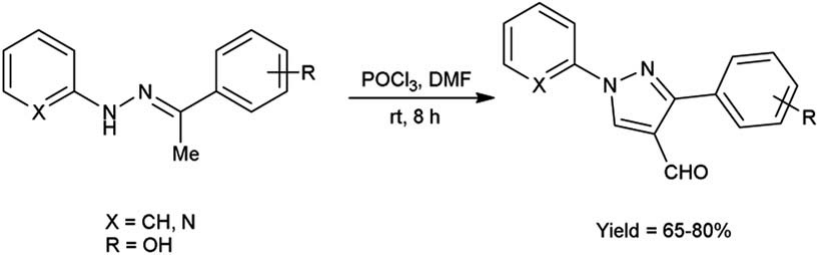
Synthesis of 3-aryl-4-formyl pyrazoles.

In 2016, Padalkar and co-workers^45^ synthesized few 3-aryl-4-formyl pyrazoles from substituted hydrazones using V. H. reagent (DMF-POCl_3_) by stirring the reaction mixture at room temperature for 8 hours, in good to excellent yield. Further, 2-[substituted-1*H*-pyrazol-4-yl]benzothiazoles, benzimidazoles and benzoxazoles were produced using these formyl pyrazoles and diagnosed for anti-bacterial and anti-fungal efficacy. Among the synthesized compounds, 2-[4-(1,3-benzoxazol-2-yl)-1-(pyridin-2-yl)-1*H*-pyrazol-3-yl]phenol, 5-nitro-2-[3-phenyl-1-(pyridin-2-yl)-1*H*-pyrazol-4-yl]-1*H*-benzimidazol and 2-[4-(5-nitro-1*H*-benzimidazol-2-yl)-1-(pyridin-2-yl)-1*H*-pyrazol-3-yl]phenol showed good anti-fungal activity against *Candida albicans* strains with MIC value 62.5 mg mL^−1^. Other derivatives exhibited moderate activity against *E. coli*, *S. aureus*, *C. albicans*, *A. niger* strains. Amongst the synthesized compounds, 2-[4-(1*H*-benzimidazol-2-yl)-1-(pyridin-2-yl)-1*H*-pyrazol-3-yl]phenol showed good anti-bacterial activity against *E. coli* and *S. aureus* with each having MIC value of 62.5 mg mL^−1^. The growth inhibitory activity screened against fungal and bacterial strains remains unaffected while electron-donating and electron-withdrawing substituents attached on benzothiazoles, benzimidazoles and benzoxazoles containing pyrazole moiety. The synthesized compounds exhibited significantly higher anti-bacterial activity as compared to fungal strains over analyzed microorganisms. The knowledge accrued from this investigation is expected to furnish valuable insights for the advancement of prospective pharmaceutical agents originating from benzimidazole, benzoxazole and benzothiazole compounds, with the incorporation of the pyrazole moiety, in the pursuit of pioneering anti-infective agents (Scheme 10).

**Scheme 11:**
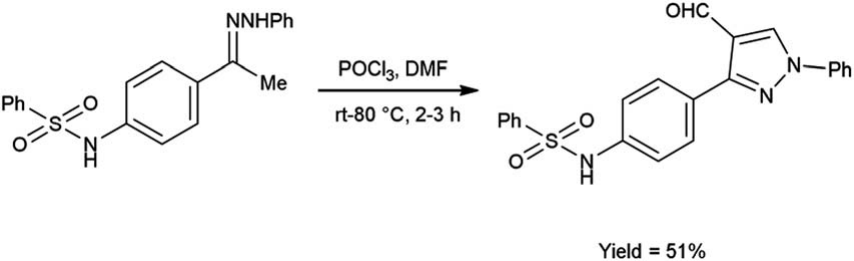
Synthesis of *N*-(4-(4-formyl-1-phenyl-1*H*-pyrazol-3-yl)phenyl)benzenesulfonamide.

In 2020, El-Mekabaty and group^46^ synthesized *N*-(4-(4-formyl-1-phenyl-1*H*-pyrazol-3-yl)phenyl)benzenesulfonamide from *N*-(4-acetylphenyl)benzenesulfonamide by using V. H. reagent by stirring the reaction mixture at room temperature for 1 hour followed by heating at 70 to 80 °C for 2 hours, in good yield. MTT colorimetric assay was used to analyse *in vitro* anti-cancer activity of obtained carbaldehyde against human breast adenocarcinoma cells (MCF-7) and human normal retina pigmented epithelium cells (RPE-1). The resulted compound exhibited cancer resistant potency with MCF-7 and RPE-1 having IC_50_ value of 21.2 ± 2.6 μM and 67.9 ± 3.5 μM respectively (Scheme 11).

**Scheme 12:**
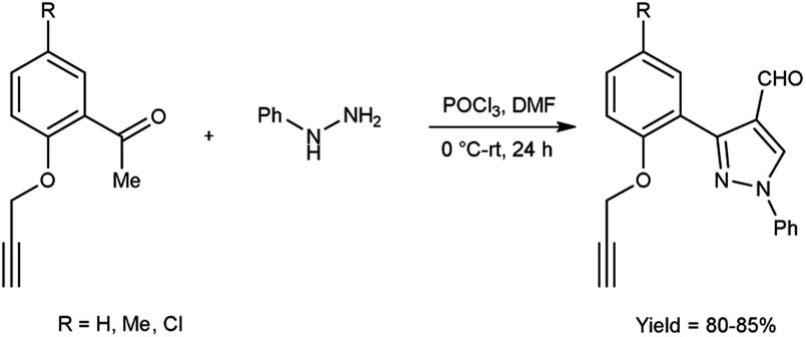
Synthesis of 1-phenyl-3-[2-(prop-2-yn-1-yloxy)-phenyl substituted]-1*H*-pyrazole-4-carbaldehyde.

In 2017, Pervaram and co-workers^47^ synthesized 1-phenyl-3-[2-(prop-2-yn-1-yloxy)-phenylsubstituted]-1*H*-pyrazole-4-carbaldehyde from 1-(5-substituted-2-(prop-2-yn-1-yloxy)phenyl)ethan-1-one and phenyl hydrazine using V. H. reagent (DMF-POCl_3_) at 0 °C for 15 minutes followed by stirring reaction mixture at room temperature for 24 hours, in excellent yield. These formyl pyrazoles were further reacted with sodium azide to form triazole-based derivatives. The synthesized compounds were evaluated for their anti-bacterial and anti-fungal activities. Amongst them, compounds 3-{2-[(1-benzyl-1*H*-1,2,3-triazol-4-yl)methoxy]-5-methylphenyl}-1-phenyl-1*H*-pyrazole-4-carbaldehyde and 3-{5-chloro-2-[(1-(3-chlorobenzyl)-1*H*-1,2,3-triazol-4-yl)methoxy]phenyl}-1-phenyl-1*H*-pyrazole-4-carbaldehyde found to be more efficient against *A. niger* and *A. flavus* fungi in comparison to standard drug nystatin (Scheme 12).

**Scheme 13:**
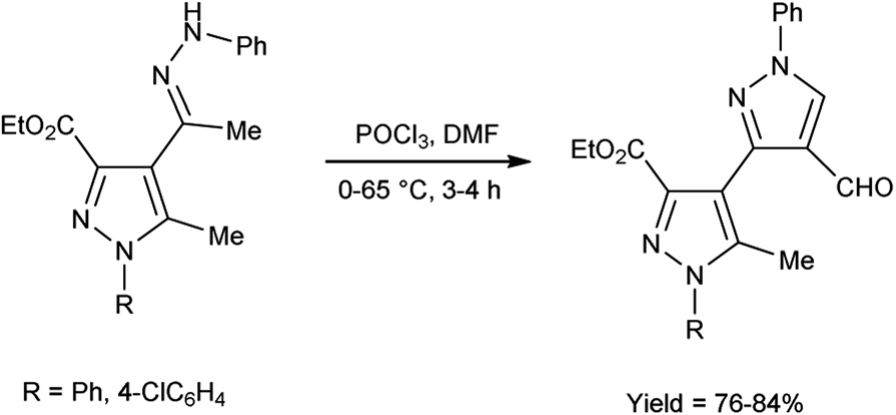
Synthesis of ethyl-1-aryl-5-methyl-4-[1-(phenylhydrazinylidene)ethyl]-1*H*-pyrazole-3-carbaldehydes.

In 2017, Potopnyk *et al.*^48^ reported the synthesis of ethyl-1-aryl-5-methyl-4-[1-(phenylhydrazinylidene)ethyl]-1*H*-pyrazole-3-carbaldehydes from ethyl(*E*)-5-methyl-1-aryl-4-(1-(2-phenylhydrazono)ethyl)-1*H*-pyrazole-3-carboxylate using V. H. reagent (DMF-POCl_3_). The reaction mixture was stirred at 0–10 °C for 10 minutes followed by stirring at room temperature for 1 hour consequently heating at 65 °C for 2–3 hours to get the desired carbaldehydes. The resultant carbaldehydes were expected to exhibit several bioactivities. Moreover, different methodology was also employed by utilizing Fischer indole reaction conditions with these hydrazones but the reaction did not proceed (Scheme 13).

**Scheme 14:**
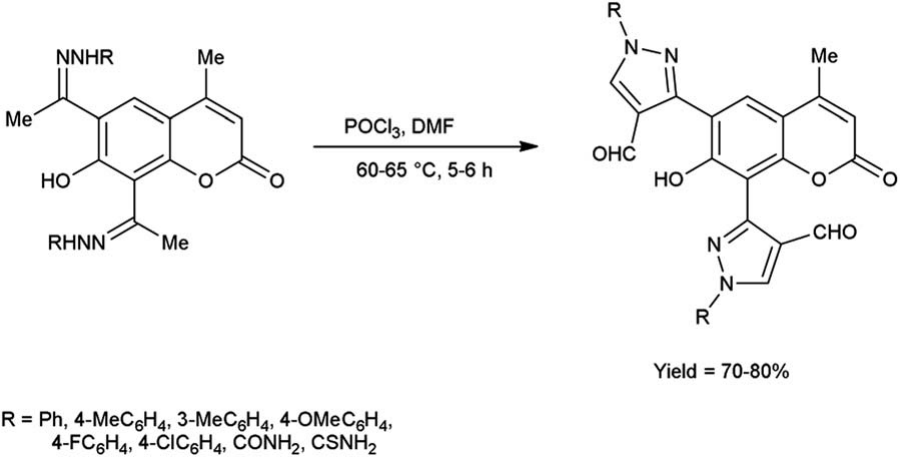
Synthesis of coumarin appended bis(formyl pyrazole) derivatives.

In 2016, Nagamallu and co-workers^49^ synthesized some new coumarin appended bis(formyl pyrazole) derivatives from the hydrazones, carbazones and thiocarbazones using V. H. reagent by stirring the reaction mixture at 60 to 65 °C for 5 to 6 hours, very good yield. Quantitative structure–activity relationship (QSAR) was employed to analyse the disease resistant potency of resultant products. Out of these, compound 3,3′-(7-hydroxy-4-methyl-2-oxo-2*H*-chromene-6,8-diyl)bis(4-formyl-1*H*-pyrazole-1-carboxamide) having minimum inhibitory concentration (MIC) value of 12.5 ± 0.81 μg mL^−1^ was found to be more potent against anti-fungal and anti-bacterial activity in contrast to standard drug fluconazole and ciprofloxacin. The compound 3,3′-(7-hydroxy-4-methyl-2-oxo-2*H*-chromene-6,8-diyl)bis(4-formyl-1*H*-pyrazole-1-carbothioamide) demonstrated more promising inhibitory effect against anti-fungal and anti-bacterial strains with MIC value of 12.5 ± 0.37 μg mL^−1^. In this study, new protocols have been developed to boost structural complexity while reducing synthetic steps to build new coumarin-based bis(formyl pyrazole) derivatives (Scheme 14).

**Scheme 15:**
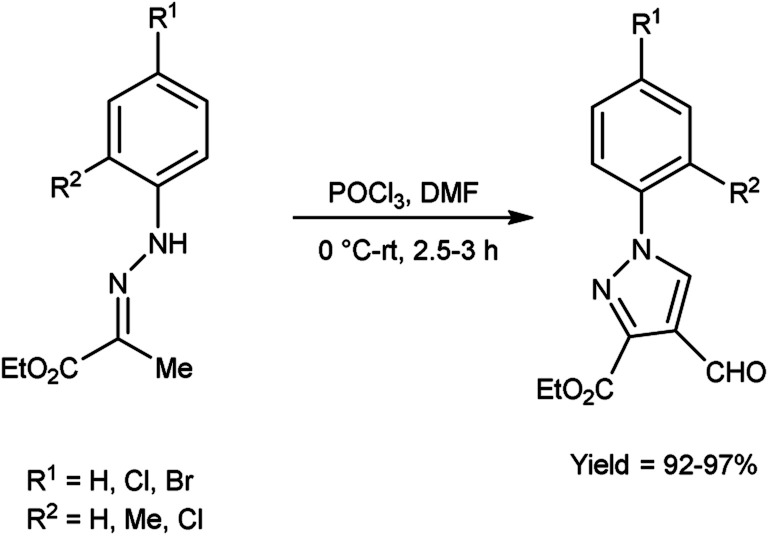
Synthesis of ethyl-4-formyl-1-substitute- phenyl-1*H*-pyrazole-3-carboxylate derivatives.

In 2016, Swami *et al.*^50^ synthesized ethyl-4-formyl-1-substituted-phenyl-1*H*-pyrazole-3-carbaldehydes derivatives in excellent yield when substituted phenyl hydrazones were reacted with V. H. reagent in cold condition and followed by stirring the reaction mixture at room temperature for about 3 hours. These formyl pyrazoles were employed as substrate in the multicomponent reaction for producing chemically and medicinally important pyrazole-coupled imidazo[1,2-*a*]pyridine. This is a straightforward, effectual, safer and environment friendly approach for the synthesis of pyrazole-coupled imidazo-pyridine (Scheme 15).

**Scheme 16:**
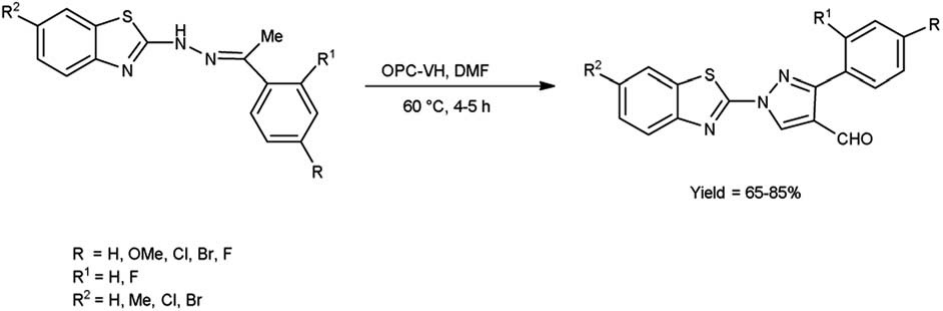
Synthesis of benzothiazole based 4-formylpyrazoles derivatives.

In 2018, Bala and co-workers^51^ synthesized benzothiazole based 4-formylpyrazoles from the corresponding hydrazones using OPC-V. H. reagent (phthaloyl dichloride and DMF) in dioxane, the reaction mixture was blended at 60 °C for 4 to 5 hours. Broth macro dilution assay was used to analyse anti-microbial activity and DPPH radical scavenging assay was employed for analysis of anti-oxidant activity of the synthesized compounds. Among the synthesized compounds, 1-(6-bromobenzo[*d*]thiazol-2-yl)-3-phenyl-1*Hs*-pyrazole-4-carbaldehyde showed promising anti-oxidant activity among all assessed compounds when compared to the standard drug ampicillin. According to the structure–activity relationship, the examined compounds were not found to be more effective against bacterial and fungal strains than conventional anti-microbial drugs (Scheme 16).

**Scheme 17:**
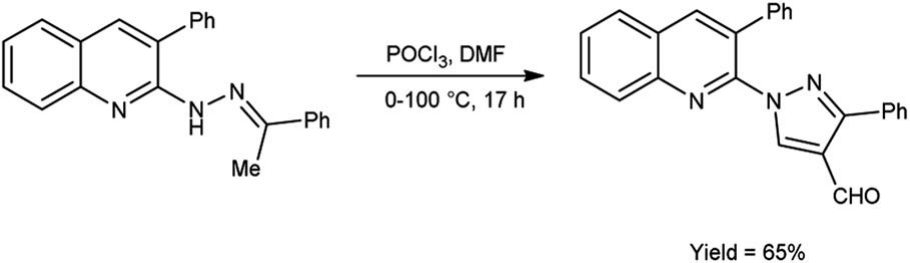
Synthesis of 3-phenyl-1-(3-phenylquinolin-2-yl)-1*H*-pyrazole-4-carbaldehyde.

In 2016, Sathiyaseelan *et al.*^52^ synthesized 3-phenyl-1-(3-phenylquinolin-2-yl)-1*H*-pyrazole-4-carbaldehyde from 3-phenyl-2-(2-(1-phenylethylidene)hydrazinyl)quinoline using V. H. reagent (DMF-POCl_3_) with stirring in ice-bath, later at room temperature for 15 minutes followed by heating the reaction mixture at water bath for 17 hours, in good yield. The utilization of cost-effective precursors, coupled with simplified work-up procedures, constitutes a significant synthetic approach for the synthesis of formyl pyrazoles (Scheme 17).

**Scheme 18:**
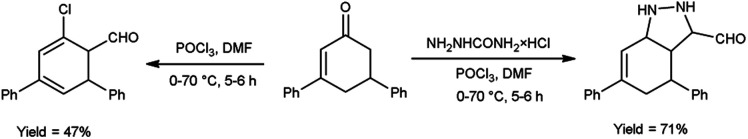
Synthesis of 4,6-diphenyl-3*a*,5-dihydro-4*H*-indazole-3-carbaldehyde and 5′-chloro-1′,2′,3′,4′-tetrahydro-[1,1′:3′,1′′-terphenyl]-4′-carbaldehyde.

In 2015, Allah and group^53^ developed an efficient protocol for the preparation of 4,6-diphenyl-3*a*,5-dihydro-4*H*-indazole-3-carbaldehyde and 5′-chloro-1′,2′,3′,4′-tetrahydro-[1,1′:3′,1′′-terphenyl]-4′-carbaldehyde from 3,5-diphenylcyclohex-2-en-1-one using V. H. reagent in ice-bath for 10–15 minutes and stirred at room temperature for 1 hour followed by heating the reaction mixture at 70 °C for 5 hours, in moderate to good yield. These compounds served as substrate in the production of benzo-thiazepines, pentahydroxyhexylidene, indazole and *N*-thiazines. The resulted products were found to have low activities against Gram-negative bacteria as compared to conventional drug chloramphenicol, but they displayed high anti-bacterial action against Gram-positive bacteria (Scheme 18).

**Scheme 19:**
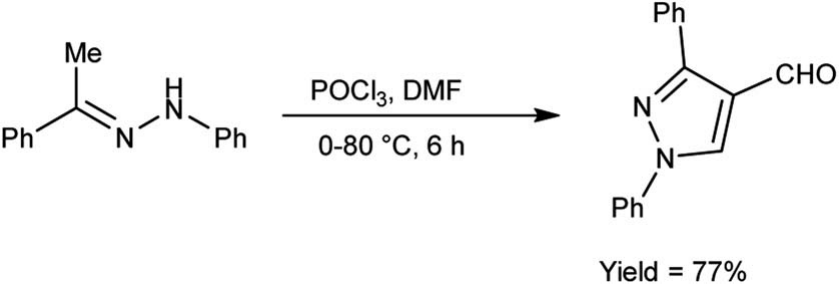
Synthesis of 1,3-diphenylpyrazole-4-carboxaldehyde.

In 2020, Shetty *et al.*^54^ produced 1,3-diphenylpyrazole-4-carboxaldehyde from phenylhydrazone using V. H. reagent (DMF-POCl_3_) in ice-bath followed by stirring the reaction mixture at 70 to 80 °C for 6 hours, in good yield. This carbaldehyde was used as an excellent precursor for the synthesis of Schiff base by reacting with primary amines. The compound, (*E*)-*N*-((1,3-diphenyl-1*H*-pyrazol-4-yl)methylene)-4-nitroaniline displayed higher anti-bacterial activity with MIC value of 17.11 ± 0.5 μg mL^−1^ against *S. aureus* and 18.85 ± 0.5 μg mL^−1^ against *E. coli* respectively when compared against standard drug ciprofloxacin. The product, (*E*)-*N*-((1,3-diphenyl-1*H*-pyrazol-4-yl)methylene)-3-nitroaniline exhibited good anti-cancer activity with MIC value 35.98 μg μL^−1^ against A459 lung cancer cell line and 47.99 μg μL^−1^ against MCF-7 cell line respectively. *In vitro*, *in silico* and SAR studies demonstrated that strong electron-withdrawing groups (NO_2_) at para and meta position of aromatic ring displayed significant anti-bacterial, anti-fungal and anti-cancer properties (Scheme 19).

**Scheme 20:**
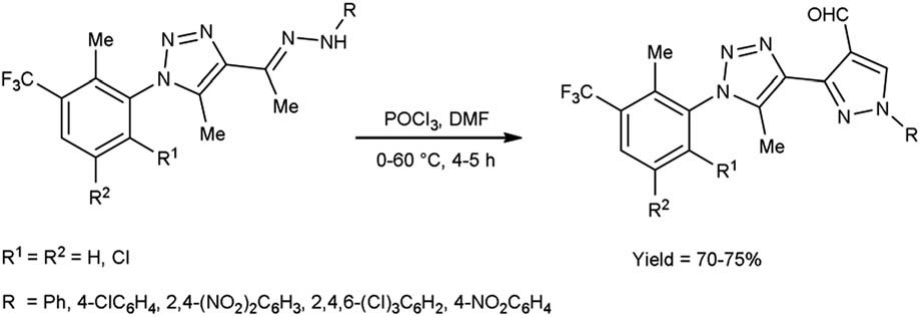
Synthesis of 1*H*-1,2,3-triazol-4-yl-1-(aryl)-1*H*-pyrazole-4-carbaldehydes.

In 2016, Bhat and co-workers^55^ provided an efficient approach to access 3-{5-methyl-1-[2-methyl-3-(trifluoromethyl)phenyl/substitutedphenyl]-1*H*-1,2,3-triazol-4-yl}-1-(aryl)-1*H*-pyrazole-4-carbaldehyde from 4-{(1*E*)-1-[2-(aryl)hydrazinylidene]ethyl}-5-methyl-1-[2-methyl-3-(trifluoromethyl)phenyl/substituted phenyl]-1*H*-1,2,3-triazole using V. H. reagent (DMF-POCl_3_) with stirring in cold condition for 30 minutes in inert atmosphere followed by heating at 60 °C for 4 to 5 hours, in very good yield. The recently synthesized compounds underwent screening to evaluate their *in vitro* anti-oxidant, anti-fungal and anti-bacterial activities. Based on the structure–activity correlation, compounds containing 8-trifluoromethyl quinyl, phenyl, 2,4-dinitro phenyl, 2,4,6-trichloro phenyl and pthalazinyl substituents on the pyrazole ring were exhibited higher anti-bacterial efficacy. Similarly, the compounds containing the substituents *i.e.*, 2-methyl-3-trifuoromethyl-5,6-dihydrophenyl, 2-methyl-3-trifuoromethyl-5,6-dichloro at first position of triazole ring and phenyl, pthalazinyl on the second position of pyrazole ring displayed potent radical scavenging with IC_50_ value ranging from 14.47 to 19.23 mM as compared to standard drug ascorbic acid with an IC_50_ value of 12.27 mM (Scheme 20).

**Scheme 21:**
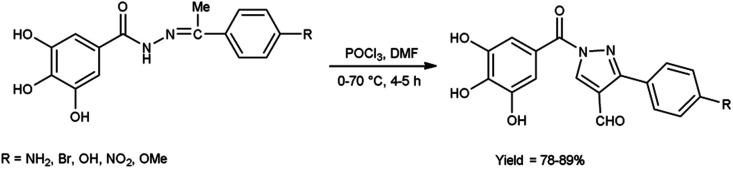
Synthesis of 3-substituted phenyl-5-(3,4,5-trihydroxyphenyl)-4*H*-pyrazole-4-carbaldehydes.

In 2023, Nashaan and co-workers^56^ reported the synthesis of 3-substituted phenyl-5-(3,4,5-trihydroxyphenyl)-4*H*-pyrazole-4-carbaldehyde from (*Z*)-3,4,5-trihydroxy-*N*′-(1-(4-substituted phenyl)ethylidene)benzohydrazide using V. H. reagent blended in ice-bath followed by heating at 70 °C for 4 to 5 hours, in excellent yield. These carbaldehydes were screened by using agar well diffusion method against two bacterial strains *Klebsiella pneumonia* and *S. aureus*. These scaffolds were demonstrated an excellent anti-bacterial activity in contrast to standard drug ampicillin (Scheme 21).

**Scheme 22:**
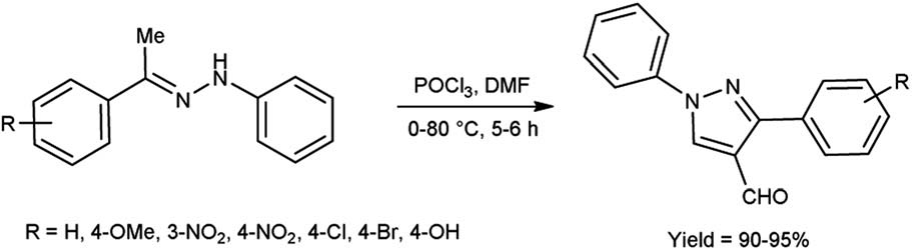
Synthesis of 1-phenyl-3-substitutedphenyl-1*H*-pyrazole-4-carbaldehydes.

In 2022, Mamatha *et al.*^57^ prepared 1-methyl-3-substitutedphenyl-1*H*-pyrazole-4-carbaldehyde from corresponding hydrazones using V. H. reagent while stirring in ice-bath for 15 minutes followed by heating at 80 °C for 5 to 6 hours, in excellent yield. Further, these formyl pyrazoles derivatives were utilized to prepare pyrazole-conjugated benzothiazole derivatives. These Schiff bases were analysed for their anti-tubercular and anti-cancer activity by means of MTT assay and molecular docking studies. *In vitro*, compound 2-{2-[(3-(4-methoxyphenyl)-1-phenyl-1*H*-pyrazol-5-yl)methylene] hydrazinyl}methoxybenzo[*d*]thiazole showed anti-tubercular and anti-cancer activities with IC_50_ values against the HELA and KB cell lines were found to be 67.65 μg mL^−1^ and 70.31 μg mL^−1^, respectively. Evaluated products were found to be active and have least toxicity towards normal cell lines even at higher concentration with IC_50_ value >192 μg mL^−1^ (Scheme 22).

### 2.2 Formylation of pyrazole

**Scheme 23:**
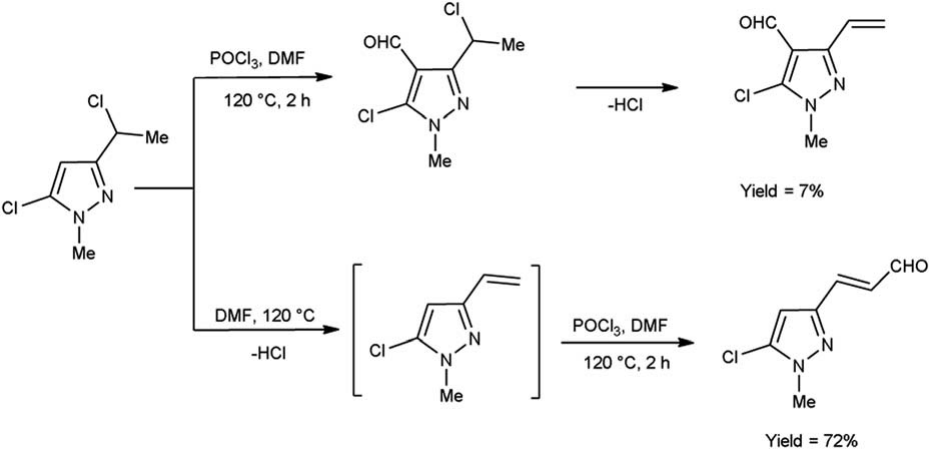
Synthesis of 5-chloro-1*H*-pyrazole-4-carbaldehyde.

In 2019, Popov and group^58^ synthesized 5-chloro-1*H*-pyrazole-4-carbaldehyde from 1,3-disubstituted-5-chloro-1*H*-pyrazoles using V. H. reagent on stirring at 0 °C for 10–15 minutes followed by heating at 120 °C for 2 hours, in good yield. The ease of implementation, convenient accessibility of initial substances and significance of the resulting pyrazoles render this protocol fascinating to researchers. Conversion of simple molecule to bioactive formyl derivatives were regarded as helpful synthons in organic and medicinal chemistry (Scheme 23).

**Scheme 24:**
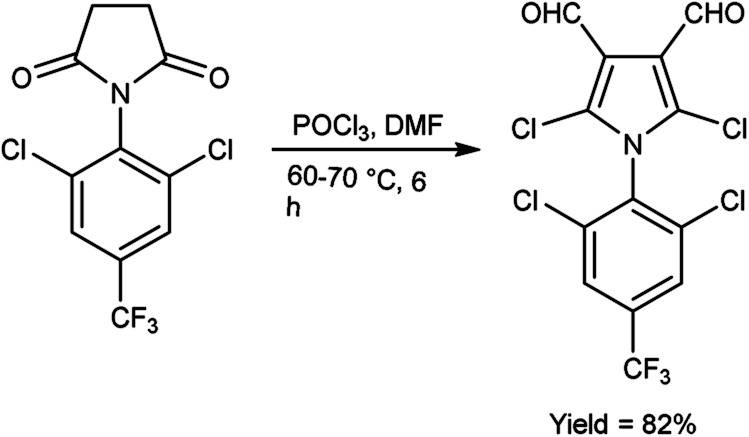
2,6-Dichloro-1-(2,6-dichloro-4-triflouromethyl-phenyl)-1,4-dihydro-pyridine-3,5-dicarbaldehyde.

In 2015, Rajput and co-workers^59^ described the preparation 2,6-dichloro-1-(2,6-dichloro-4-triflouromethyl-phenyl)-1,4-dihydro-pyridine-3,5-dicarbaldehyde from 1-(2,6-dichloro-4-triflouromethyl-phenyl)-piperidine-2,6-dione utilising V. H. reagent (DMF-POCl_3_) while stirring in cold-condition followed by heating at 60 to 70 °C for 6 hours, in excellent yield. Anti-microbial activity of synthesized compounds was evaluated against *E. coli*, *C. albicans*, *B. subtilis*, *Pseudomonas aeruginosa*, *Staphylococcus aureus* and *A. niger*. Amongst them, the compound 1-(2,6-dichloro-4-trifluoromethyl-phenyl)-piperidine-2,6-dione demonstrated moderate anti-microbial activity against *P. aeruginosa* and *S. aureus*. While compound 2,5-dichloro-1-(2,6-dichloro-4-triflouromrthyl-phenyl)-1*H*-pyrrole-3,4-dicarbaldehyde exhibited a moderate level of anti-microbial activity against *P. aeruginosa* (Scheme 24).

**Scheme 25:**
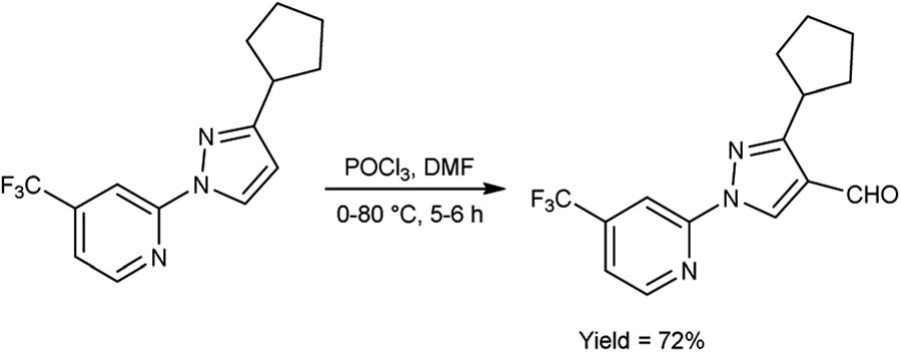
Synthesis of 3-cyclopentyl-1-(4-(trifluoromethyl)pyridin-2-yl)-1*H*-pyrazole-4-carbaldehyde.

In 2023, Pahutski and co-workers^60^ produced 3-cyclopentyl-1-(4-(trifluoromethyl)pyridin-2-yl)-1*H*-pyrazole-4-carbaldehyde from 2-(3-cyclopentyl-1*H*-pyrazol-1-yl)-4-(trifluoromethyl)pyridine using V. H. reagent with stirring at 0 °C followed by heating at 80 °C for 5 to 6 hours, in good yield. Further, these carbaldehydes were used to prepare some new *N*-arylpyrazole-4-methylpiperidines having good efficacy against a variety of lepidopteran pests. The present study proposes the possibility of a new mechanism of action, although the specific target site remains unidentified. These compounds do not appear to function as established biochemical pathways of insect control because the exact method of action is still an anonymous (Scheme 25).

### 2.3 Synthesis of miscellaneous compounds

**Scheme 26:**
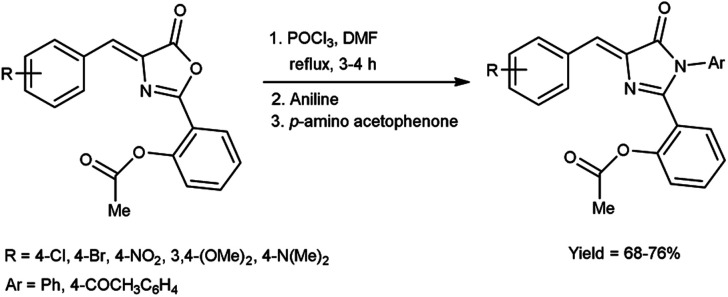
Synthesis of 2-(4-arylidene-5-oxo-1-aryl-4,5-dihydro-1*H*-imidazole-2-yl)phenyl acetates.

In 2019, Aparna and co-workers^61^ described the synthesis of 2-(4-arylidene-5-oxo-1-aryl-4,5-dihydro-1*H*-imidazole-2-yl)phenyl acetates from 2-substituted-oxo-4,5-dihydrooxazol-2-yl-phenyl acetate on treatment of V. H. reagent, aniline and 4-amino acetophenone on stirring the reaction mixture in ice-bath for 1 hour followed by refluxing for 2 to 3 hours resulted in good yield. *In vitro*, pour plate method and molecular docking studies were employed to screen the activity of synthesized compounds. Among the synthesized compounds, 2-{4-[4-(acetoxybenzylidene)-5-oxo-1-phenyl-4,5-dihydro-1*H*-imidazole-2-yl]}phenyl acetate, 2-[4-(4-bromobenzylidene)-5-oxo-1-phenyl-4,5-dihydro-1*H*-imidazole-2-yl]phenyl acetate and 2-[1-(4-acetylphenyl)-4-(4-chlorobenzylidene)-5-oxo-4,5-dihydro-1*H*-imidazole-2-yl]phenyl acetate exhibited high anti-bacterial potency against *Klebsiella pneumonia*, *B. subtilis*, *E. coli* and *S. aureus*. The resulting products also act as precursor for the synthesis of several amino acids as well as drugs (Scheme 26).

**Scheme 27:**
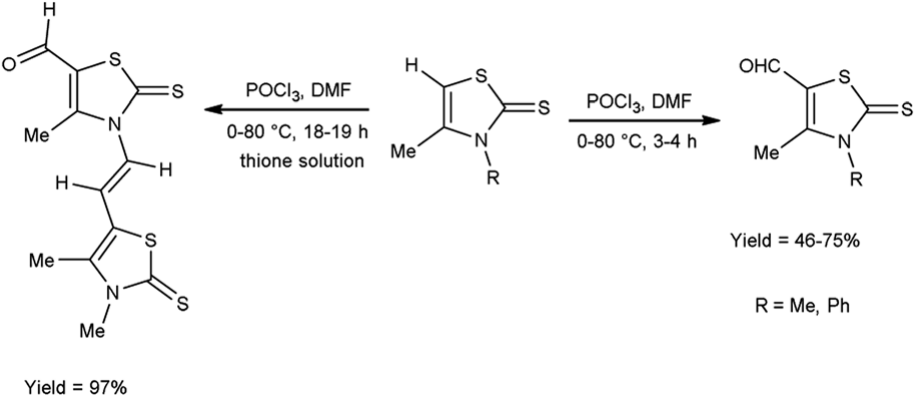
Synthesis of thiazolinethione-5-carbaldehydes.

In 2018, Soud *et al.*^62^ synthesized few thiazolinethione-5-carbaldehyde from 4-thiazolinethione using V. H. reagent in ice-bath and further stirring the reaction mixture at room temperature for 1 hour followed by heating at 80 °C for 3 to 18 hours, in good to excellent yield. The resulting products could be served as crucial building blocks in the synthesis of a variety of heterocycles exhibited potent biological activity and have good predictions for usage in optoelectronics (Scheme 27).

**Scheme 28:**
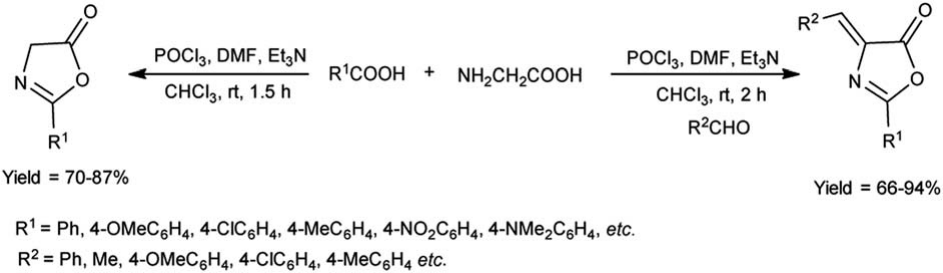
Synthesis of oxazolones.

In 2018, Fadavi *et al.*^63^ developed a new protocol for the preparation of oxazol-5-ones (oxazolones) and 4-arylmethylene-oxazolones through the cyclization of *N*-acyl-α-amino acids using V. H. reagent (DMF-POCl_3_), triethylamine in dry chloroform by stirring at room temperature for 1.5 to 2 hours. The resulting compounds were obtained in good to excellent yield in one pot. Notably, this approach boasts the advantages of operating under mild reaction conditions, delivering good to excellent yield of products and offering simplified procedures (Scheme 28).

**Scheme 29:**
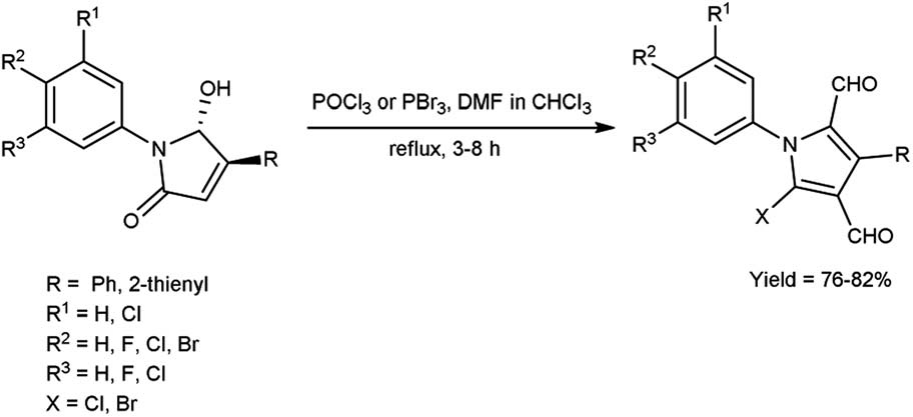
Synthesis of diformylated-*N*-arylpyrroles.

In 2015, Barman *et al.*^64^ prepared some diformylated-*N*-arylpyrroles from *trans*-1,4-diaryl-5-hydroxypyrrolidin-2-ones using V. H. reagent (DMF-POCl_3_) in chloroform at 0 °C and stirred the reaction mixture at room temperature for 40 minutes followed by refluxing for 3 to 8 hours, in good to excellent yield. The operational ease and economic feasibility of this approach, along with the safety, straightforwardness, low cost certainly lead to the method's widespread adoption for possible outcomes of further investigation (Scheme 29).

**Scheme 30:**
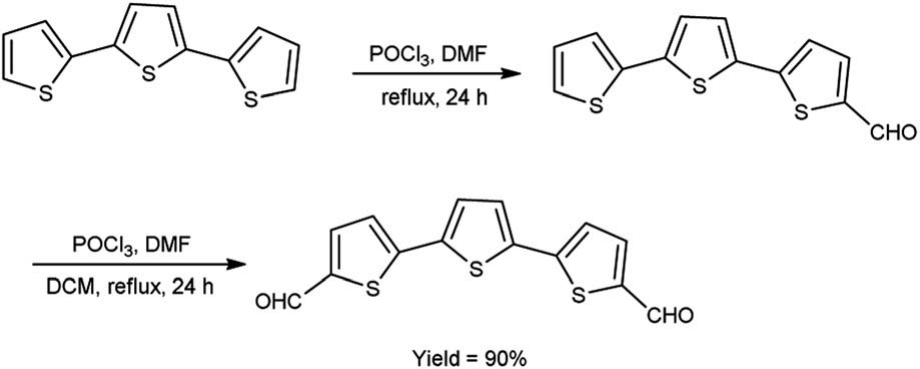
Synthesis of 5,5′′-diformyl-2,2′:5′,2′′-terthiophene.

In 2021, Dmour *et al.*^65^ provided the synthesis of 5,5′′-diformyl-2,2′:5′,2′′-terthiophene from 2,2′:5′,2′′-terthiophene on treatment with V. H. reagent in dry chloroform at 0 °C and stirred at 40 °C followed by refluxing for 24 hours, in excellent yield. The compound, [2,2′:5′,2′′:5′′,2′′quaterthiophene]-5-cyanoacrylic acid was obtained from 5,5′′-diformyl-2,2′:5′,2′′-terthiophene and used to form dye-sensitized solar cells (DSSCs) that have the potential for low-cost fabrication. The resulting dyes have high power conversion efficiency and low current density when compared to other dyes (Scheme 30).

**Scheme 31:**
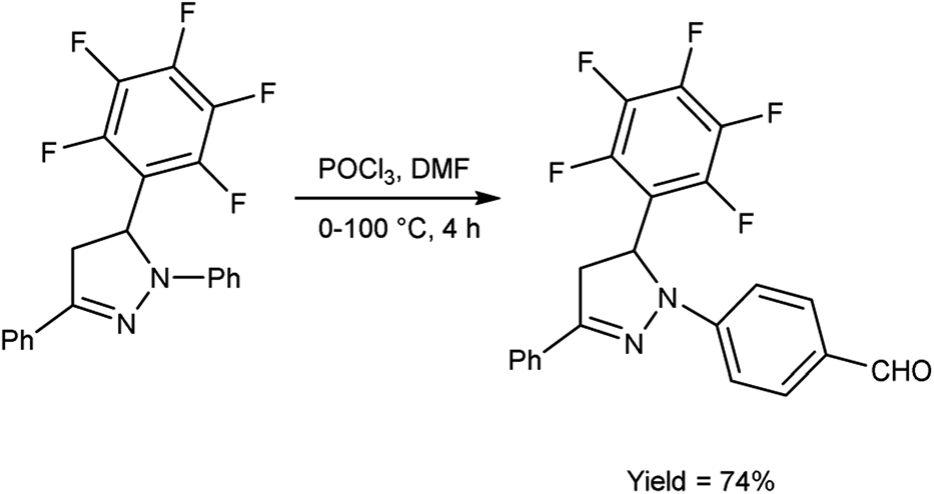
Synthesis of [3-(pentafluorophenyl)-5-phenyl-4,5-dihysdro-1*H*-pyrazol-1-yl]benzaldehyde.

In 2019, Shelkovnikov *et al.*^66^ synthesized some [3-(pentafluorophenyl)-5-phenyl-4,5-dihydro-1*H*-pyrazol-1-yl]benzaldehyde from dihydropyrazole using V. H. reagent at 0 °C followed by stirring the reaction mixture at 90 to 100 °C for 4 hours, in good yield. The polyfluorinated triphenyl-4,5-dihydro-1*H*-pyrazoles containing different amines were produced by using [3-(pentafluorophenyl)-5-phenyl-4,5-dihydro-1*H*-pyrazol-1-yl]benzaldehyde used as precursors for producing donor–acceptor dyes to generate nonlinear electro-optics chromophores with abundant possibilities for future customization (Scheme 31).

## 3. Synthesis of six-membered heterocyclic compounds

### 3.1 Synthesis of pyridine carbaldehyde

**Fig. 6:**
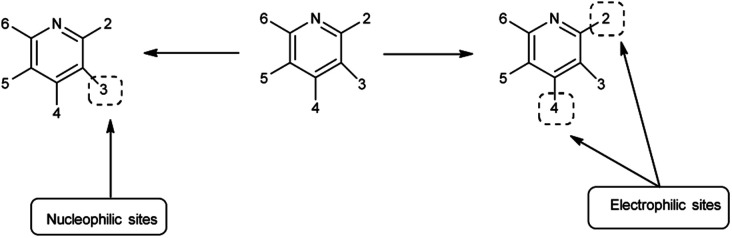
Pyridine.

Pyridine^67^ is a basic heterocyclic organic compound with the chemical formula C_5_H_5_N. Due to the presence of an electronegative nitrogen atom this ring becomes electron deficient. Unlike benzene derivatives, it is less reactive towards electrophilic aromatic substitutions (Fig. 6).

**Fig. 7:**
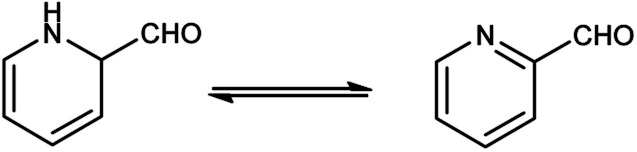
Tautomer's of pyridine carbaldehyde.

**Fig. 8:**
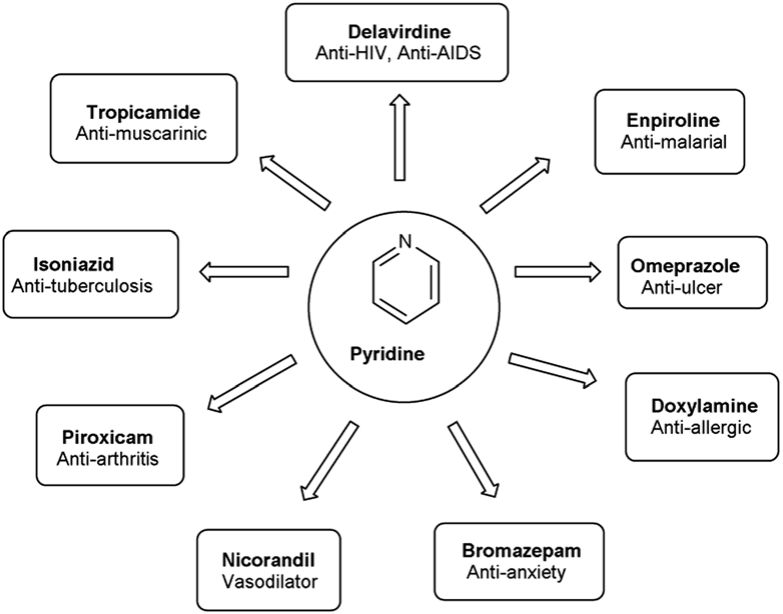
Pyridine based drugs.

Pyridine carbaldehyde exist in tautomeric form as shown in Fig. 7. The strong organometallic bases can readily metalates pyridine, suggesting it is more susceptible to nucleophilic substitutions. In pharmacology, it is widely used as anti-bacterial, anti-viral, anti-histamine, anti-allergic, anti-bactericide and as herbicide (Fig. 8).

**Scheme 32:**
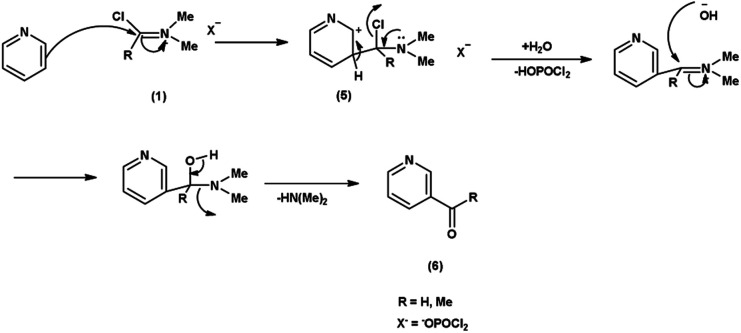
Plausible mechanism for formylation of pyridine ring.

In the first step, the reaction of DMF-POCl_3_ generates an iminium salt (1). The electron rich pyridine approaches the electron deficient carbon of iminium salt (1) generating an intermediate (5). Further, the basic hydrolysis of this intermediate (5) results in the formation of the formylated pyridine derivative (6). The resonance stabilization of the iminium ion augments its reactivity, ensuring efficient formylation^68^ (Scheme 32).

**Scheme 33:**
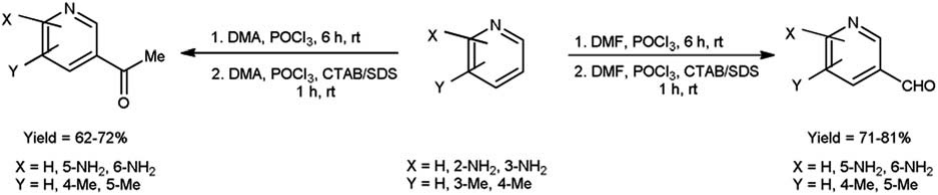
Synthesis of pyridine carbaldehyde.

In 2022, Tasneem *et al.*^68^ showed a significant drain in reaction time in micellar media by using V. H. reagent in formylation and acetylation in pyridine. This reaction follows second order kinetics when {[V. H. reagent] = [Substrate]} and pseudo first order kinetics when {[Substrate] ≫ [V. H. reagent]}. Rate of this reaction increased in micellar condition and time was brought down from 6 hours to 1 hour since micelles act as catalyst in the reaction. The reaction afforded the synthesis of desired compounds in good to excellent yield. The majority of previously documented approaches have been characterized by the implementation of rigorous reaction conditions, leading to challenges in the subsequent workup process. In light of this, the utilization of micelles has afforded the opportunity to establish a rapid and dynamic methodology for the facile synthesis of formyl and acetyl derivatives of pyridine, thus presenting potent analogues with significant potential in terms of pharmacological activities (Scheme 33).

**Scheme 34:**
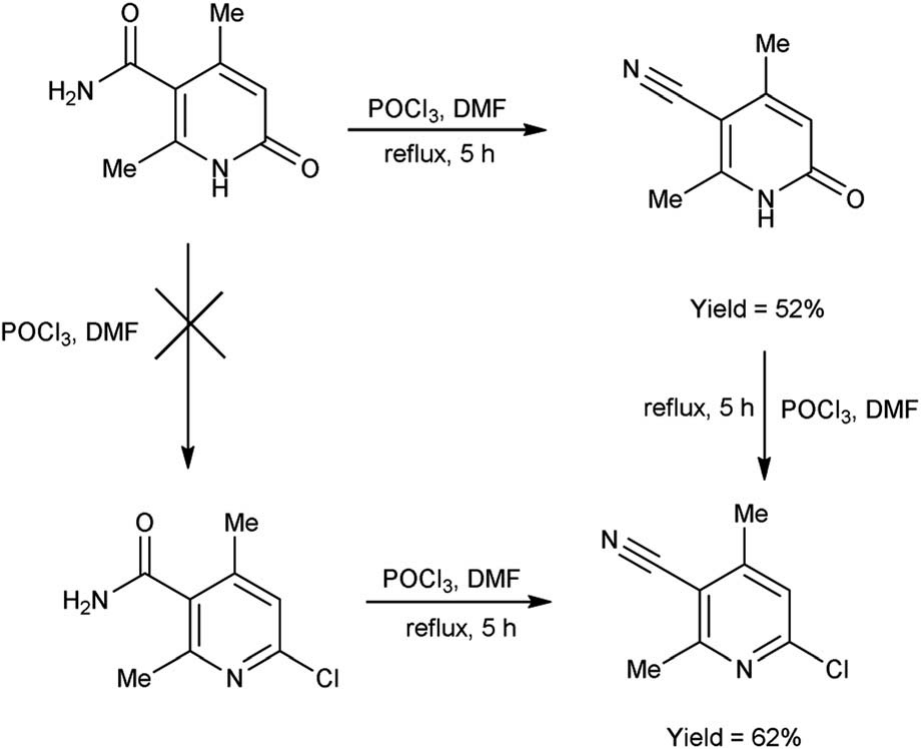
Synthesis of 2-chloro-4,6-dimethylnicotinonitrile.

In 2017, Koval *et al.*^69^ synthesized some new 2-chloro-4,6-dimethylnicotinonitrile from 2,4-dimethyl-6-oxo-1,6-dihydropyridine-3-carboxamide using V. H. reagent (DMF-POCl_3_) by refluxing the reaction mixture for 5 hours. Plausible synthetic route for the production of novel pyridines through sequential transformations was simulated by density functional theory (DFT). The desired products were obtained in good yield and expected to show some biological properties (Scheme 34).

### 3.2 Synthesis of miscellaneous compounds

**Scheme 35:**
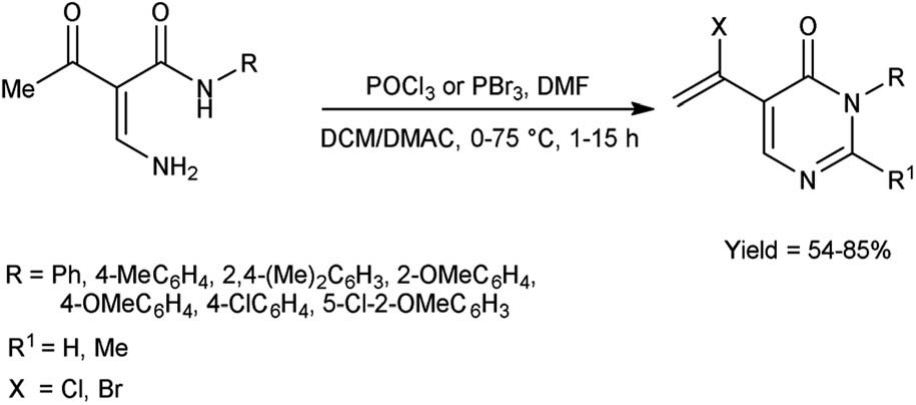
Synthesis of pyrimidin-4-(3*H*)-ones.

In 2011, Zhang *et al.*^70^ synthesized few pyrimidin-4-(3*H*)-ones in excellent yield from amino propenamides using V. H. reagent at 0 °C followed by heating at 75 °C for 2 hours in which formylation, halogenation and intramolecular nucleophilic cyclization reactions occurs. The slow conversion was happened when the reaction performed at 60 °C during optimization and even not enhanced by further addition of POCl_3_. The experimental studies have indicated the ideal conditions for synthesizing pyrimidin-4-(3*H*)-ones involve using 2.5 equivalents of POCl_3_ with 1.5 equivalents of DMF in DCM. This approach attracts attention due to easy implementation, uses commonly accessible substrates, requires only moderate conditions, and may produce a diverse array of potentially valuable compounds (Scheme 35).

**Scheme 36:**
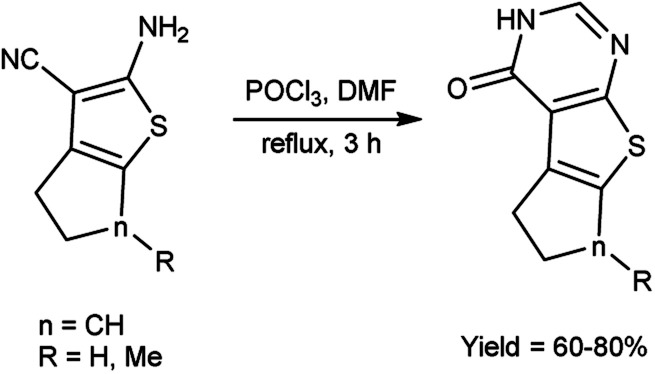
Synthesis of thieno[2,3-*d*]pyrimidin-4(3*H*)-one derivatives.

In 2020, Datoussaid *et al.*^71^ synthesized few thieno[2,3-*d*]pyrimidin-4(3*H*)-one derivatives from 2-amino-3-cyanothiophene using V. H. reagent (DMF-POCl_3_) by stirring the reaction mixture at room temperature for 3 hours followed by refluxing, in good to excellent yield. The current study outlines the development of a straightforward, expeditious, and efficacious method for synthesizing a variety of thieno[2,3-*d*]pyrimidin-4(3*H*)-ones (Scheme 36).

**Scheme 37:**
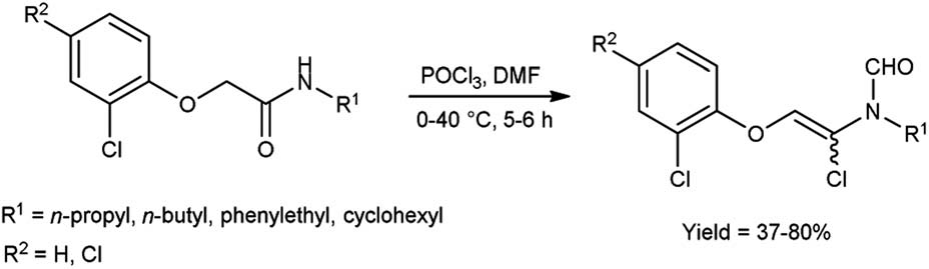
Synthesis of *Z*/*E N*-(1-chlorovinyl)formamides.

In 2019, Tang *et al.*^72^ synthesized some *Z*/*E N*-(1-chlorovinyl)formamides from 2-phenoxyethanamides using V. H. reagent in ice-bath for 1 hour followed by stirring at 40 °C for 5 hours, in moderate to excellent yield. The introduced C-α-chloro increased the versatility of enamides due to C-α position and phenoxy substitutions at C-β position of *N*-vinylformamides and *Z*/*E* isomers of *N*-vinylformamides (NVF). It is anticipated that this methodology can be expanded to facilitate the production of a broader range of functionalized NVFs. Additionally, the potential of this approach is expected to be relevant for synthesis of various substrates (Scheme 37).

**Scheme 38:**
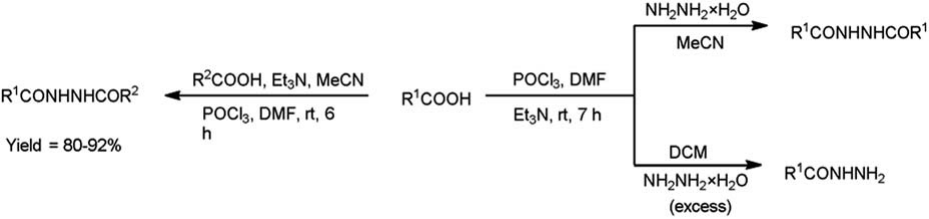
Synthesis of diacylhydrazines.

In 2017, Zarei *et al.*^73^ synthesized some symmetrical and unsymmetrical diacylhydrazines from carboxylic acid using V. H. reagent in dry chloroform or acetonitrile at room temperature for 6 to 13 hours, in good to excellent yield. The V. H. reagent is a practical and efficient reagent utilized for the synthesis of acylhydrazines. The simplicity and effectiveness of this method makes it suitable for implementation in large-scale manufacturing process (Scheme 38).

**Scheme 39:**
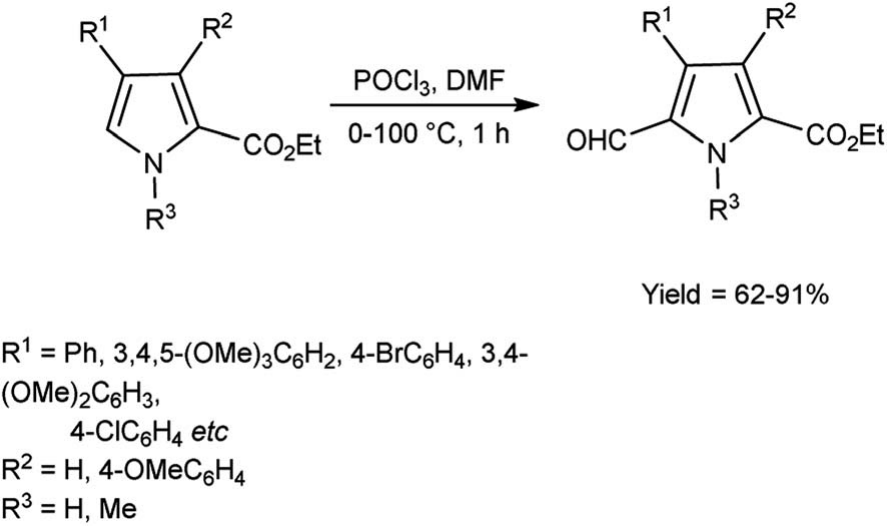
Synthesis of carbethoxypyrroles.

In 2008, Gupton *et al.*^74^ efficiently synthesized some pyrrole carbaldehyde derivatives from substituted pyrrole ring using microwave accelerated V. H. reagent in ice-bath for 45 minutes followed by heating at 100 °C in microwave reactor for 14 minutes, in good to excellent yield. These formylated pyrroles are an efficient and flexible precursor for natural products bearing pyrrole ring *viz.* permethylstorniamide A and polycitones A and B. These formylation reactions offer an effective, adaptable and regiocontrolled technology for the synthesis of significant class of natural compounds. These processes should also provide quick access to a diverse spectrum of highly functionalized pyrroles for further physiologically prompted SAR investigations (Scheme 39).

**Scheme 40:**
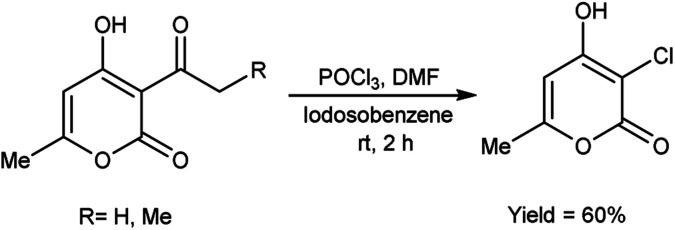
Synthesis of 3-chloro-4-hydroxy-6-methyl-2*H*-pyran-2-ones.

In 2005, Prakash *et al.*^75^ prepared 3-chloro-4-hydroxy-6-methyl-2*H*-pyran-2-one by stirring 3-acetyl-4-hydroxy-6-methyl-2*H*-pyran-2-one using V. H. reagent in combination with iodosobenzene at room temperature for 2 hours, in average yield. The hydroxy group at position-4 remains intact while the C–C bond in ketone was cleaved during the reaction. The outcomes of this investigation have engendered a keen interest in the comprehensive elucidation of the prospective utilities arising from the synergistic interaction between IOB (iodine(iii)bis(trifluoroacetate)) and V. H. reagent (Scheme 40).

**Scheme 41:**
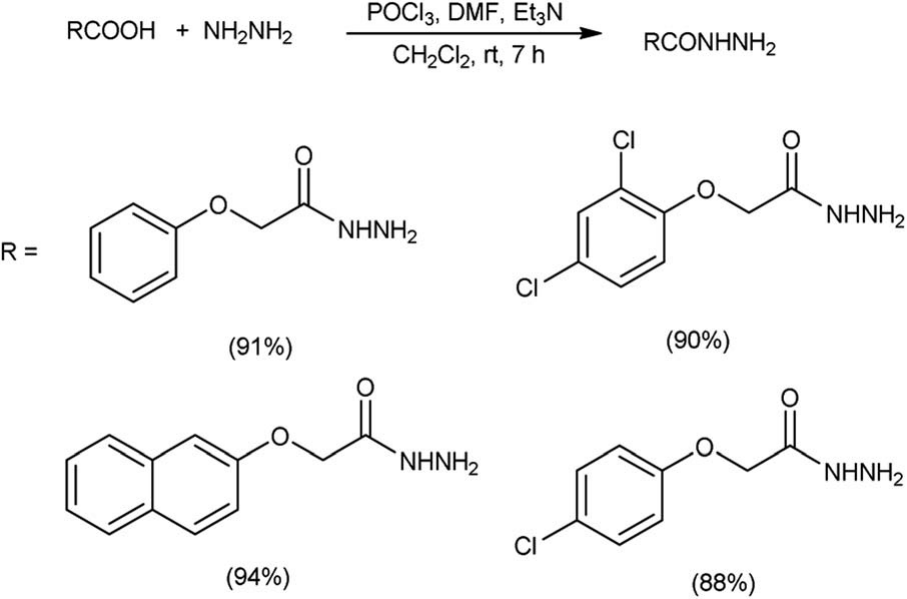
Synthesis of acylhydrazines.

In 2017, Zarei and co-workers^76^ synthesized few symmetrical and unsymmetrical acylhydrazines from carboxylic acid with the help of V. H. reagent by stirring the reaction mixture at room temperature for 7 hours, in excellent yield. The implementation of the V. H. reagent obviates the necessity to handle and synthesize acyl halides, rendering the method remarkably efficient, especially for large-scale applications, as the by-products can be readily separated and removed through simple aqueous work-up procedures (Scheme 41).

## 4. Synthesis of fused-rings heterocyclic compounds

### 4.1 Synthesis of quinoline carbaldehyde

**Fig. 9:**
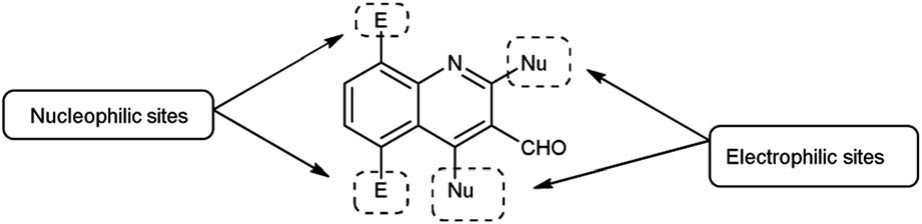
Quinoline.

**Fig. 10:**
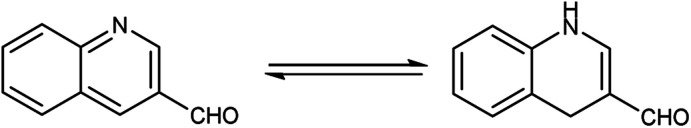
Tautomer's of quinoline carbaldehyde.

Quinoline,^77^ also known as 1-azanaphthalene or benzo[*b*]pyridine, is an aromatic heterocyclic nitrogen-containing compound with the molecular formula C_9_H_7_N. It reacts similar to benzene and pyridine as it is a weak tertiary base and forms salts with acids. It participates in both nucleophilic and electrophilic substitution reactions (Fig. 9). Tautomeric structures of quinoline carbaldehyde are outlined in Fig. 10.

**Fig. 11:**
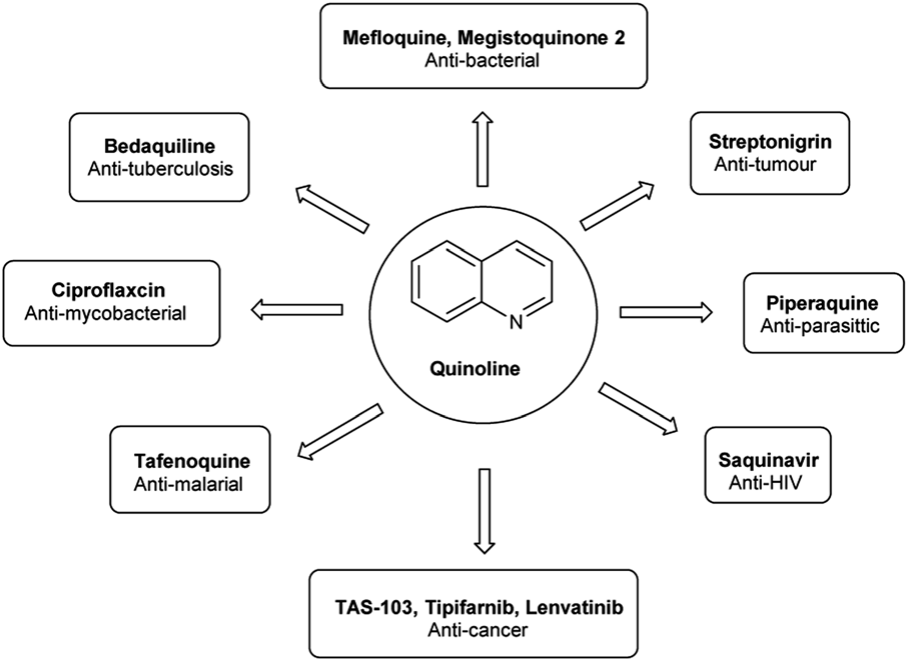
Quinoline based drugs available in the market.

It is a common component of different natural products (*e.g.*, cinchona alkaloids) and pharmacological research has shown that numerous substances with different biological effects also contain the quinolone ring system. The anti-bacterial, anti-fungal, anti-malarial, anthelmintic, anti-spasmodic, cardiotonic, anti-inflammatory and analgesic properties of quinoline are discovered as shown in Fig. 11.

**Scheme 42:**
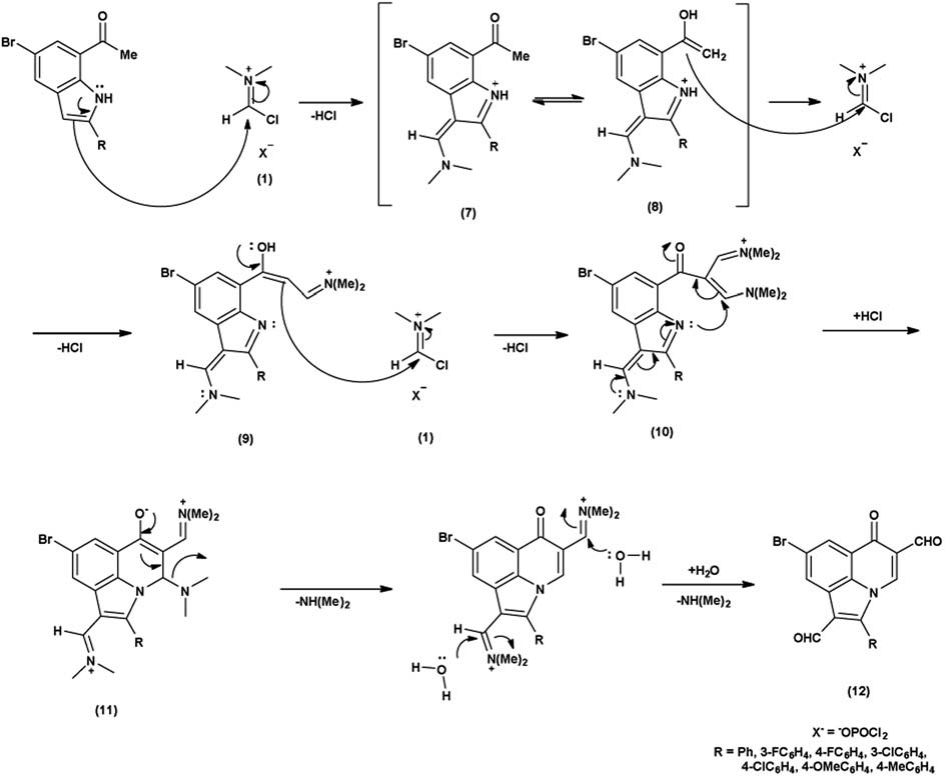
Plausible mechanism for formylation of indole ring.

In the initial step, the mechanism involves the attack on V. H. reagent (1) through C-3 of the indole of the substrate to form resonance stabilized iminium intermediate (7). The intermediate (7) containing methyl group, depicted in its mesomeric enol form (8). The ensuing enol tautomer's C=C bond attack on iminium salt (1), thereby yielding the intermediate (9). This process is akin to the prior mechanism as above. This intermediate acts as a nucleophile (9), executing another attack on the iminium salt (1) to produce intermediate (10). Further, the reaction of intermediate (10) through cyclization and subsequent release of dimethylamine to produce intermediate (11) takes place. The hydrolysis of the intermediate (11) executed under basic conditions lead to the isolation of final compound (12)^78^ (Scheme 42).

**Scheme 43:**
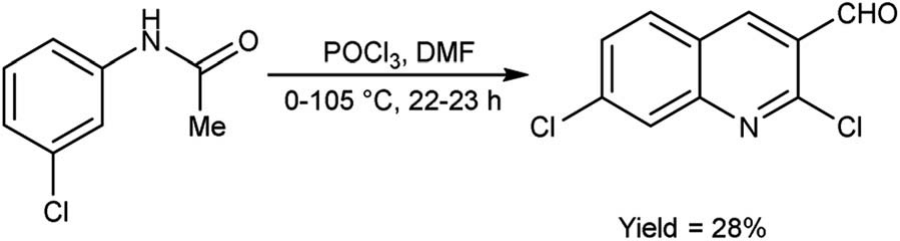
Synthesis of chloroquinoline analogue.

In 2021, Abdi and co-workers^79^ synthesized chloroquinoline from substituted phenylacetamide using V. H. reagent (DMF-POCl_3_) at 0 °C for 30 minutes followed by heating the reaction mixture at 105 °C for 22 hours, in average yield. The five different new chloroquinoline derivatives were synthesized using chloroquinoline which displayed anti-bacterial and anti-oxidant activities. Among the synthesized compounds, 7-chloro-2-ethoxyquinoline-3-carbaldehyde and 2,7-dichloroquinoline-3-carboxamide had excellent anti-bacterial activity against *E. coli*, with inhibiting regions of 12.00 ± 0.00 mm and 11.00 ± 0.04 mm respectively. The compound, 2,7-dichloroquinoline-3-carbonitrile demonstrated strong anti-bacterial action against *S. aureus* and *P. aeruginosa*, with an area of inhibition of 11.00 ± 0.03 mm as compared to amoxicillin (180.00 mm). On the other hand, compound 7-chloro-2-methoxyquinoline-3-carbaldehyde was found to be active against *S. pyogenes*, with an area of inhibition of 11.00 ± 0.02 mm. According to the molecular study, 7-chloro-2-ethoxyquinoline-3-carbaldehyde may be deemed as an effective molecule for future investigation as an anti-bacterial and anti-cancer medication (Scheme 43).

**Scheme 44:**
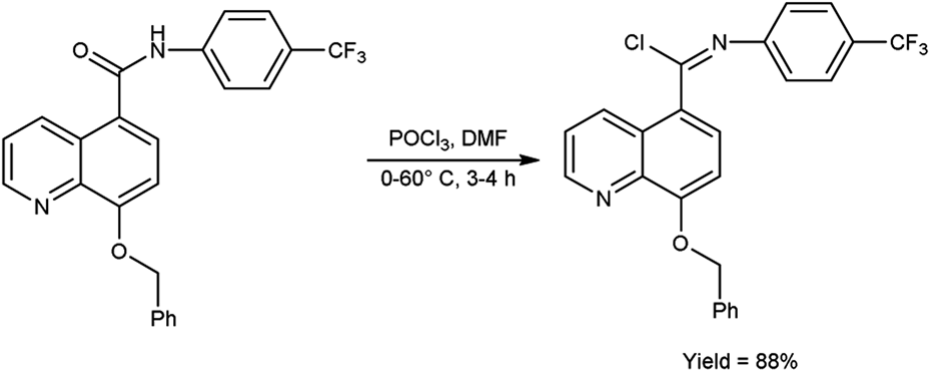
(*E*)-8-(Benzyloxy)-*N*-(4-(trifluoromethyl)phenyl)quinoline-5-carbimidoyl chloride.

In 2017, Vellalacheruvu and co-workers^80^ synthesized (*E*)-8-(benzyloxy)-*N*-(4-(trifluoromethyl)phenyl)quinoline-5-carbimidoyl chloride from 8-(benzyloxy)-*N*-(4-(trifluoromethyl)phenyl)quinoline-5-carboxamide using V. H. reagent (DMF-POCl_3_) at 0 °C, then stirred the reaction mixture at room temperature for 1 hour followed by heating at 60 °C for 3 hours, in excellent yield. The tetrazole derivatives of carbamate and urea were produced using the synthesized compound as a precursor by conventional method. This strategic circumvention involved the utilization of anhydrous toluene as the solvent medium, ensuring an impeccably moisture-free environment for the reaction. Furthermore, the entirety of the reaction is meticulously executed within a controlled argon atmosphere (Scheme 44).

**Scheme 45:**
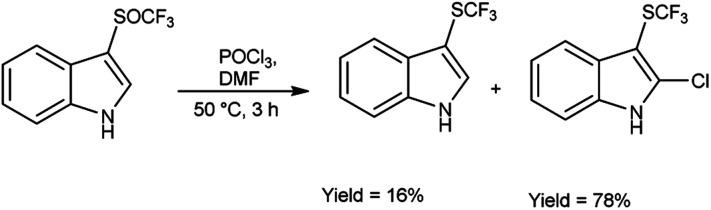
Synthesis of trifluoromethylsulfinylation.

In 2018, Wei *et al.*^81^ synthesized trifluoromethyl thiolation and biofunctionalization of indoles from sodium trifluoromethane sulfinate (CF_3_SO_2_Na) using V. H. reagent (DMF-POCl_3_) at 50 °C for 3 hours, in good yield. The method employed was devoid of metallic components, utilized a single reaction vessel, was cost-effective and employed readily accessible reagents such as phosphorus oxychloride (POCl_3_) or phosphorus oxobromide (POBr_3_), which provided an additional functional group for subsequent transformations (Scheme 45).

**Scheme 46:**
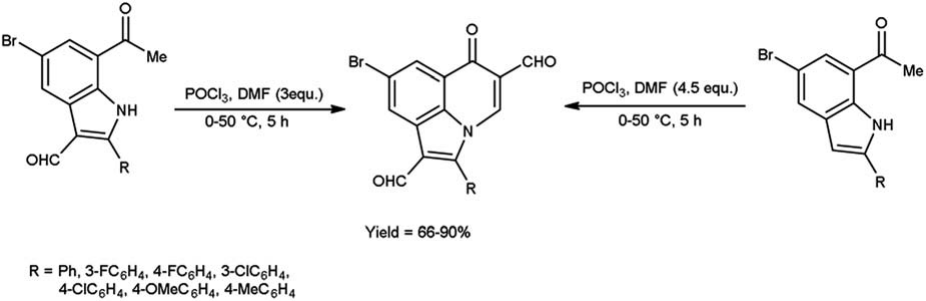
Synthesis of synthesized 8-bromo-6-oxo-2-phenyl-6*H*-pyrrolo[3,2,1-*ij*]quinoline-1,5-dicarbaldehydes.

In 2019, Mphahlele and group^78^ synthesized few 8-bromo-6-oxo-2-phenyl-6*H*-pyrrolo[3,2,1-*ij*]quinoline-1,5-dicarbaldehyde from 7-acetyl-5-bromo-2-phenyl-1*H*-indole-3-carbaldehyde using V. H. reagent and from 1-(5-bromo-2-phenyl-1*H*-indole-7-yl)ethan-1-one by using V. H. reagent (4.5 equivalents) at 0 °C for 2 hours followed by heating at 50 °C for 3 hours, in good to excellent yield. The most notable aspect of this approach is the synthesis of C–C and C–N bonds within a reaction vessel, resulting in the formation of poly-carbon-substituted pyrroloquinolines. The approach proposed in this study is an excellent tool for the identification of certain new bioactive compounds due to unique characteristics and the significance of pyrrolo[3,2,1-*ij*]quinoline analogues in pharmaceutical chemistry. The utilization of this methodology on the enolizable group *i.e.*, Schiff’s bases will result in the production of additional innovative pyrroloquinoline derivatives (Scheme 46).

**Scheme 47:**
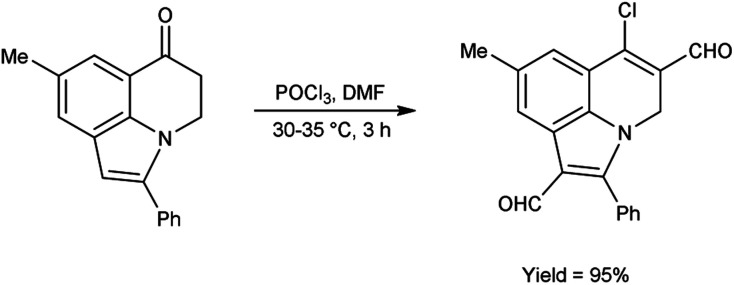
Synthesis of 6-oxo-6*H*-pyrrolo[3,2,1-*ij*]quinoline-1,5-dicarbaldehyde.

In 2011, Layek and co-workers^82^ prepared 6-chloro-8-methyl-2-phenyl-4*H*-pyrrolo[3,2,1-*ij*]quinoline-1,5-dicarbaldehyde from 8-methyl-2-phenyl-4,5-dihydro-6*H*-pyrrolo[3,2,1-*ij*]quinolin-6-one using V. H. reagent (DMF-POCl_3_) by stirring the reaction mixture at 30–35 °C for 3 hours, in excellent yield. The V. H. reagent was utilized to prepare the analogue based on functionalized indole framework (Scheme 47).

**Scheme 48:**
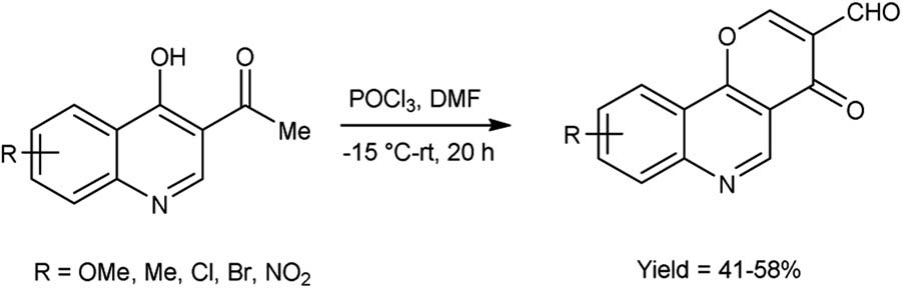
Synthesis of pyrano[3,2-*c*]quinolone-3-carbaldehydes.

In 2019, Zaman *et al.*^83^ synthesized some pyrano[3,2-*c*]quinolone-3-carbaldehydes from 3-acetyl-4-hydroxyquinoline using modified V. H. reagent at −15 to −20 °C and stirred the reaction mixture at room temperature for 20 hours, in moderate to good yield. These carbaldehydes were screened for their anti-bacterial and anti-fungal activity against *B. subtilis*, *E. coli* and *S. aureus* using the disc diffusion method. Among the synthesized compounds, 9-bromo-4-oxo-4*H*-pyrano[3,2-*c*]quinoline-3-carbaldehyde displayed maximum anti-bacterial activity against *E. coli* with inhibition zone of 14.2 ± 1.2 mm, whereas compound, 7-methyl-4-oxo-4*H*-pyrano[3,2-*c*]quinoline-3-carbaldehyde exhibited good anti-fungal activity against *S. Aureus* and compound 8-chloro-4-oxo-4H-pyrano[3,2-*c*]quinoline-3-carbaldehyde against *B. subtilis* with inhibition zone of 9.7 ± 1.2 mm and 13.25 ± 0.5 mm respectively when compared with standard drug rifampicin. The highest anti-fungal activity was shown in case of compound 9-bromo-4-oxo-4*H*-pyrano[3,2-*c*]quinoline-3-carbaldehyde against A. flavus with inhibition zone of 13.2 ± 1.0 mm while compound 7-methyl-4-oxo-4*H*-pyrano[3,2-*c*]quinoline-3-carbaldehyde displayed good anti-fungal activity against *A. niger* and compound 8-methoxy-4-oxo-4*H*-pyrano[3,2-*c*]quinoline-3-carbaldehyde against *A. alternata* with inhibition zone of 9.5 ± 0.2 mm and 8.2 ± 0.5 mm respectively when compared with reference drug anti-biotic fluconazole. This reaction exemplifies a one-pot synthesis methodology. Moreover, the resultant product exhibited a high potential for facilitating the chain reactions (Scheme 48).

**Scheme 49:**
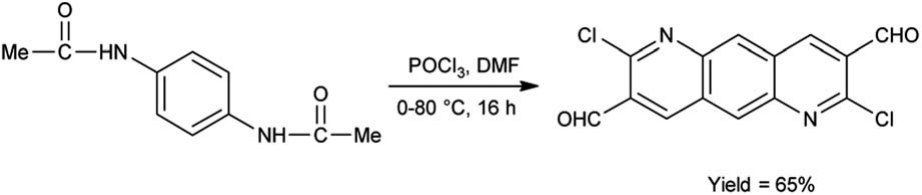
Synthesis of 2,7-dichloropyrido[2,3-*g*]quinoline-3,8-dicarbaldehyde.

In 2022, Thakafy and co-workers^84^ synthesized 2,7-dichloropyrido[2,3-*g*]quinoline-3,8-dicarbaldehyde from *p*-phenylenediamine using V. H. reagent (DMF and POCl_3_) in ice-bath and stirred at 80 °C for 16 hours, in good yield. This carbaldehyde was used as a precursor for the synthesis of chalcones. Further, the resulting chalcones were condensed with mesalazine to produce coloured azo dye. The resulting compound showed maximum absorption at 450 nm by using spectrophotometer. Beer's law was obeyed over the 0.5–27.5 μg mL^−1^ concentration range with a molar absorptivity 9494.37 mol cm^−1^. The limit of detection and quantitation is 0.161 and 0.538 μg mL^−1^. The recovery was 101.48% with a relative standard deviation ≤3.265% (Scheme 49).

### 4.2 Synthesis of imidazo-pyrimidine carbaldehyde

**Fig. 12:**
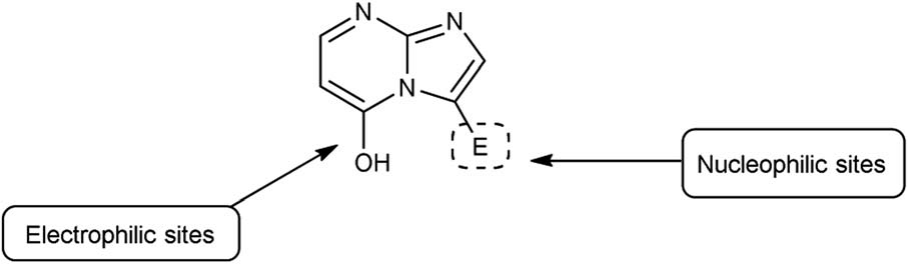
Imidazo-pyrimidine.

**Fig. 13:**
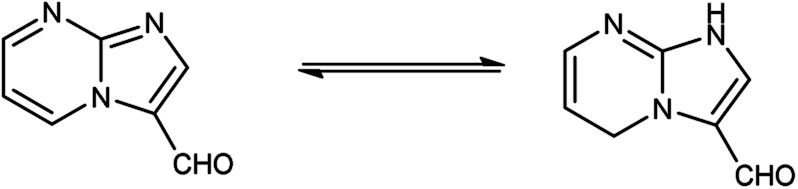
Tautomer's of imidazo-pyrimidine carbaldehyde.

**Fig. 14:**
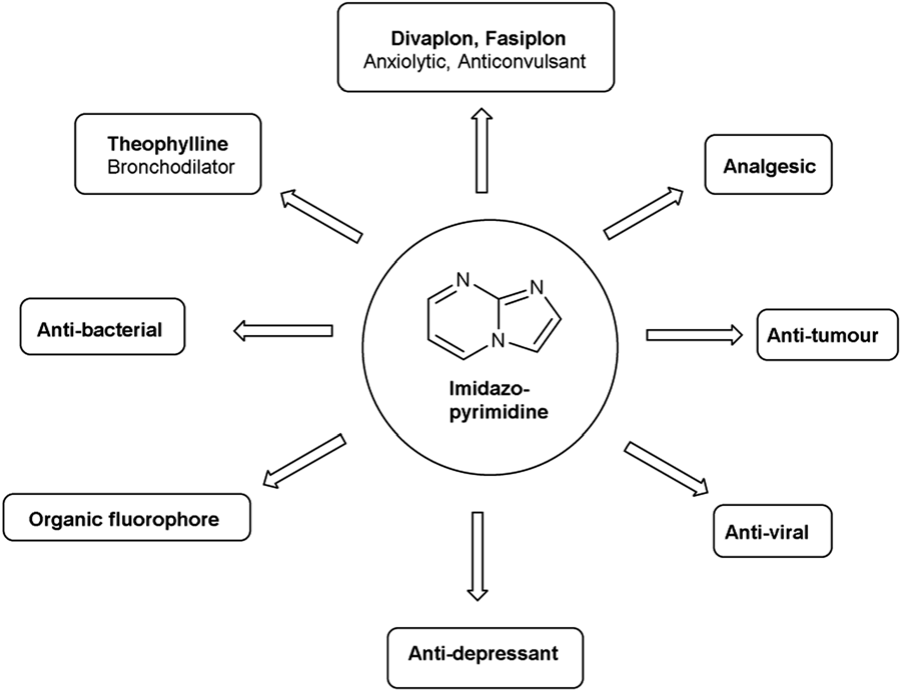
Biological profile of imidazo-pyrimidines.

Imidazo-pyrimidine^85^ is a bicyclic heterocycle with a six-membered pyrimidine ring joined by a five-membered imidazole ring having both nucleophilic and electrophilic sites (Fig. 12), exist in different tautomeric forms as shown (Fig. 13). These bicyclic ring systems have been reported to have anti-bacterial, anti-tumor, anti-analgesic, anti-viral and anti-depressant activities in literature (Fig. 14).

**Scheme 50:**
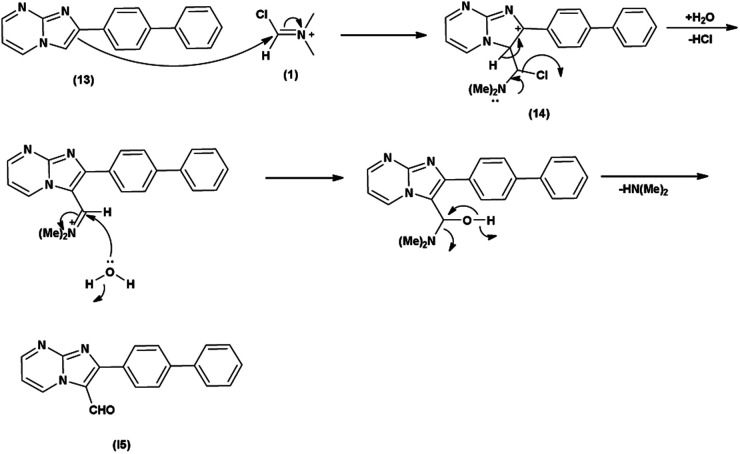
Plausible mechanism for formylation of imidazo-pyrimidine.

Initially, the formation of an iminium salt (1) from V. H. reagent takes place. Subsequently, the imidazole-pyrimidine ring (13) partakes in a nucleophilic attack on the iminium salt (1) thereby generating an intermediate (14). The hydrolytic workup of this intermediate (14) culminates in the formylation of imidazo-pyrimidine ring (15). The intricate resonance-enhanced nature of the iminium species greatly accentuates its reactivity, ensuring the efficacy of the formylation process^86^ (Scheme 50).

**Scheme 51:**
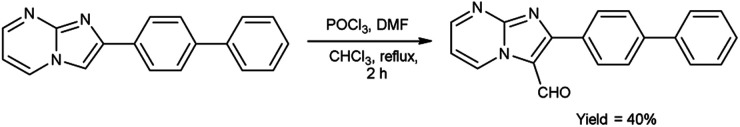
Synthesis of 2-biphenylimidazo[1,2-*a*]pyrimidine-3-carbaldehyde.

In 2018, Naeemah *et al.*^86^ synthesized some new 2-biphenyl-imidazo[1,2-*a*]pyrimidine-3-carbaldehyde from 2-biphenyl-imidazo(1,2-*a*)pyrimidine using V. H. reagent (DMF-POCl_3_) in chloroform at 0–5 °C followed by refluxing for 2 hours, in moderate yield. This carbaldehyde was used as precursor for the synthesis of novel 3-aminomethyl-2-biphenyl-imidazo(1,2-*a*)pyrimidine derivatives. These compounds were screened against various microorganisms to determine their efficacy as anti-bacterial agents. Amongst the synthesized compounds, 2-biphenyl-imidazo(1,2-*a*)pyrimidine, 2-biphenyl-imidazo[1,2-*a*]pyrimidine-3-carbaldehyde exhibited high activity against *E. coli*, while compounds (*E*)-1-(2-([1,1′-biphenyl]-4-yl)imidazo[1,2-*a*]pyrimidin-3-yl)-*N*-(2,3-dimethylphenyl)methanimine and (*E*)-1-(2-([1,1′-biphenyl]-4-yl)imidazo[1,2-*a*]pyrimidin-3-yl)-*N*-(3,4-dimethylphenyl)methanimine displayed good anti-bacterial activity against bacteria *S. aureus* (Scheme 51).

### 4.3 Synthesis of imidazo-pyridine carbaldehyde

**Fig. 15:**
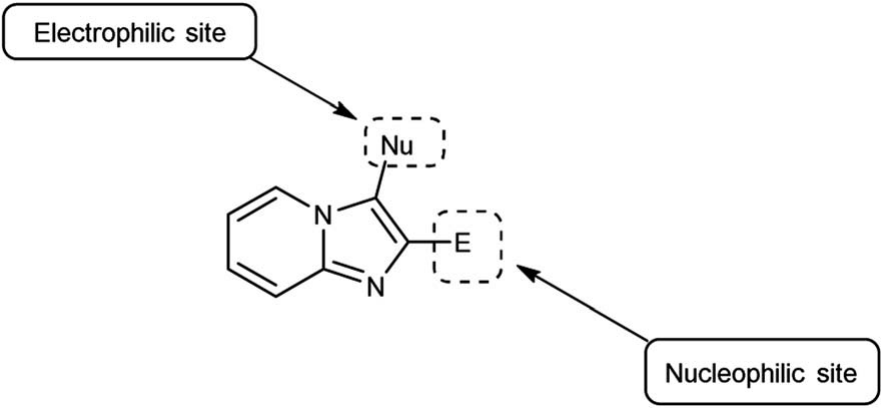
Structure of imidazo-pyridine.

**Fig. 16:**
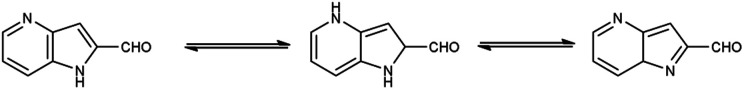
Tautomer's of imidazo-pyridine carbaldehyde.

**Fig. 17:**
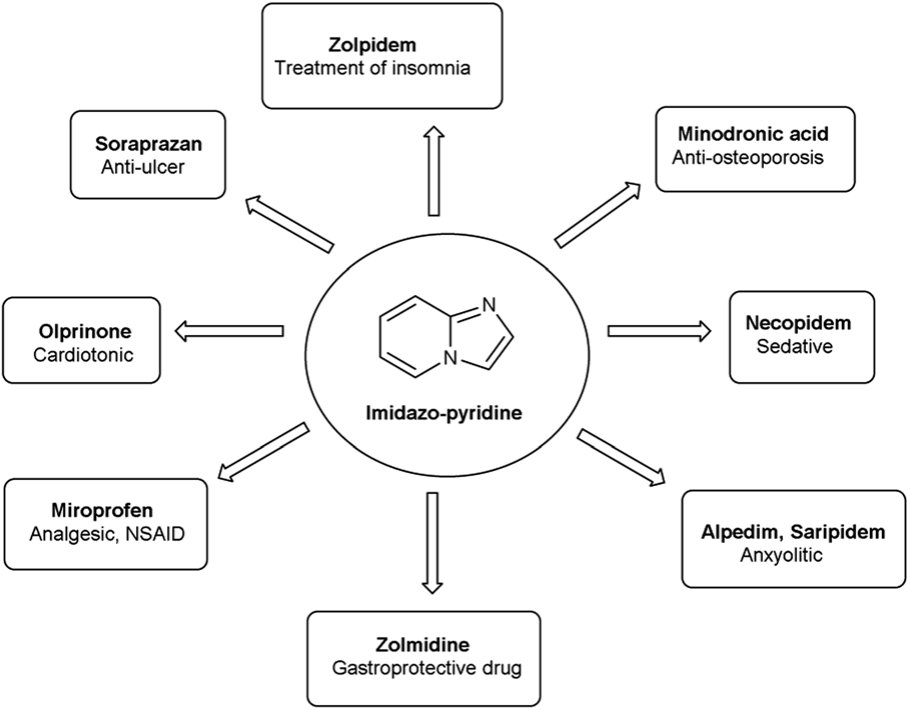
Imidazo-pyridine based drugs.

Imidazo-pyridine^87^ is a nitrogen containing bicyclic heterocycle with a five-membered imidazole ring joined by a six-membered pyridine ring including nucleophilic and electrophilic sites for substitution reactions (Fig. 15). The different tautomeric forms have been shown in Fig. 16. In addition to existing chemotherapeutic medicines, a wide range of imidazo-pyridine derivatives have been synthesized as possible anti-cancer, anti-diabetic, anti-tubercular, anti-microbial, anti-viral, anti-inflammatory agents (Fig. 17).

**Scheme 52:**
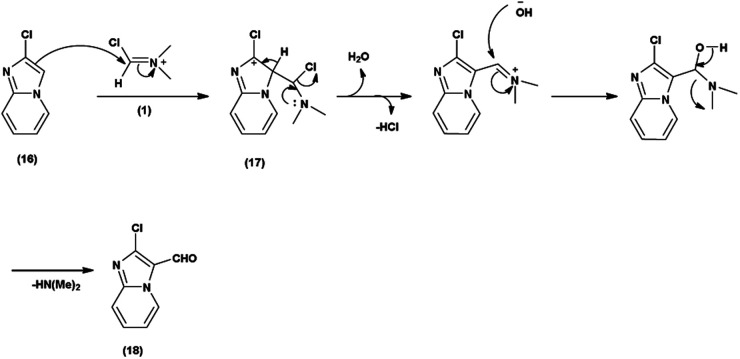
Plausible mechanism of formylation of imidazo-pyridine ring.

Primarily, the combination of V. H. reagent (DMF-POCl_3_) produces the iminium salt (1). The formation of intermediate (17) through nucleophilic attack of the imidazo-pyridine ring (16) on the electrophilic iminium salt (1) in a subsequent step takes place, which yielded the formylated product (18) upon hydrolysis. This mechanistic pathway demonstrated the ability of V. H. reagent to introduce a formyl group into the fused heterocyclic imidazo-pyridine hybrids^88^ (Scheme 52).

**Scheme 53:**
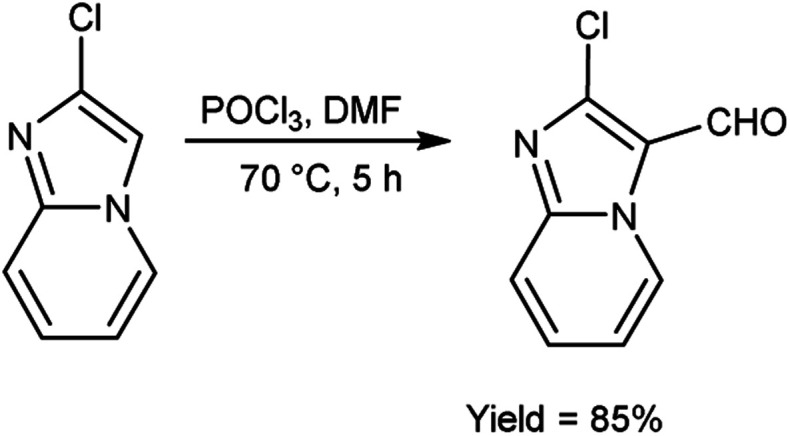
Synthesis of 2-chloroimidazo[1,2-*a*]pyridine-3-carbaldehyde.

In 2018, Antuf *et al.*^88^ synthesized 2-chloroimidazo[1,2-*a*]pyridine-3-carbaldehyde from 2-chloroimidazo[1,2-*a*]pyridine using V. H. reagent at 70 °C for 5 hours, in excellent yield. Further, synthesis of 2-indolyl-4-(2-chloroimidazo[1,2-*a*]pyridin-3-yl)- 6-ferrocenylpyrimidine have been done using this carbaldehyde as a precursor. The study of absorption bands with *λ*_max_ >480 nm revealed that ferrocene exhibited intramolecular charge transfer. The cyclic voltammetry analysis of 2-indolyl-4-(2-chloroimidazo[1,2-*a*]pyridin-3-yl)-6-ferrocenylpyrimidine exhibited a secondary, feeble and irreversible one-electron absorption wave. This wave is linked to the dimer of imidazo-pyridine ring that arises due to the oxidation of indole ring (Scheme 53).

**Scheme 54:**
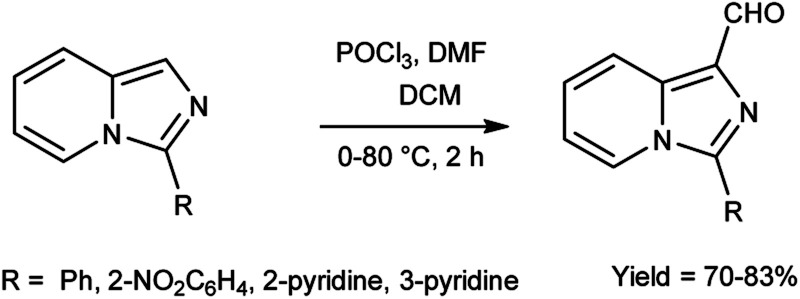
Synthesis of imidazo[1,5-*a*]pyridine carbaldehyde.

In 2015, Jinfa and group^89^ synthesized a series of 3-substituted imidazo[1,5-*a*]pyridine-1-carbaldehyde from imidazo[1,5-*a*]pyridine derivatives using V. H. reagent (DMF-POCl_3_) in ice-bath followed by stirring the reaction mixture 80 °C for 2 hours, in good yield. Further imidazo[1,5-*a*]pyridine derivatives were synthesized by using these carbaldehydes which were utilized in cell imaging, biological probes and energy conversion devices (organic light-emitting diodes, OLEDs, and organic photovoltaics (OPV). These bicyclic rings have attracted a lot of attention due to their utility in *N*-heterocyclic carbene chemistry and medical significance connected to medicines (HIV-protease and thromboxane A_2_ production inhibitors *etc.*) (Scheme 54).

### 4.4 Synthesis of pyrazolo-pyridine carbaldehyde

**Fig. 18:**
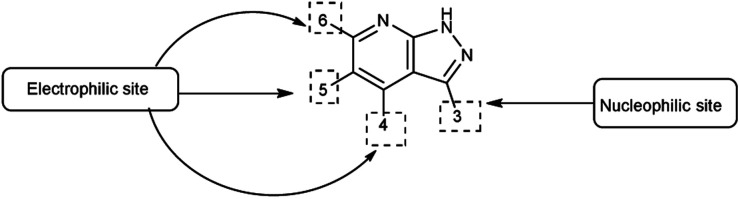
Pyrazolo-pyridine.

**Fig. 19:**
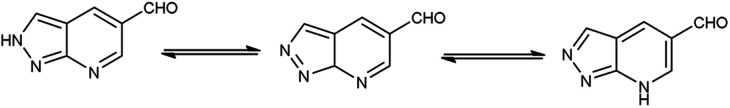
Tautomer's of pyrazolo-pyridine carbaldehyde.

**Fig. 20:**
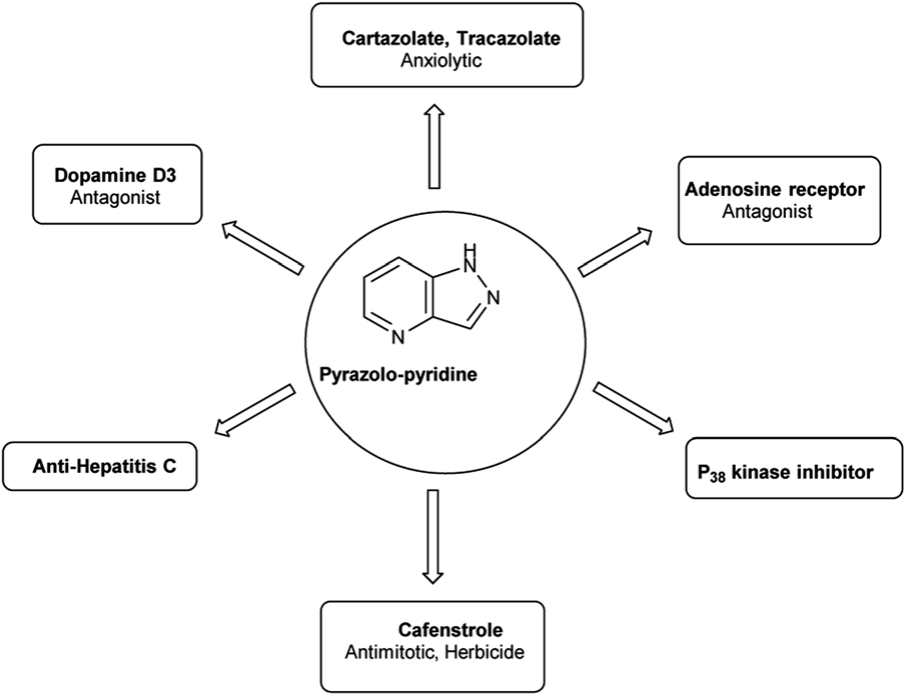
Pyrazolo-pyridine based drugs.

Pyrazolo-pyridine^90^ carbaldehyde is a class of isomeric heterocyclic compounds with the molecular formula C_6_H_5_N_3_. It is a bicyclic ring system in which pyridine ring act as nucleophilic site whereas pyrazole ring act as electrophilic site (Fig. 18). Various tautomers of these fused rings have been shown in Fig. 19. These compounds have been known to displayed several medicinal applications *i.e.*, anti-cancer, anti-hepatitis, antagonist *etc.* (Fig. 20).

**Scheme 55:**
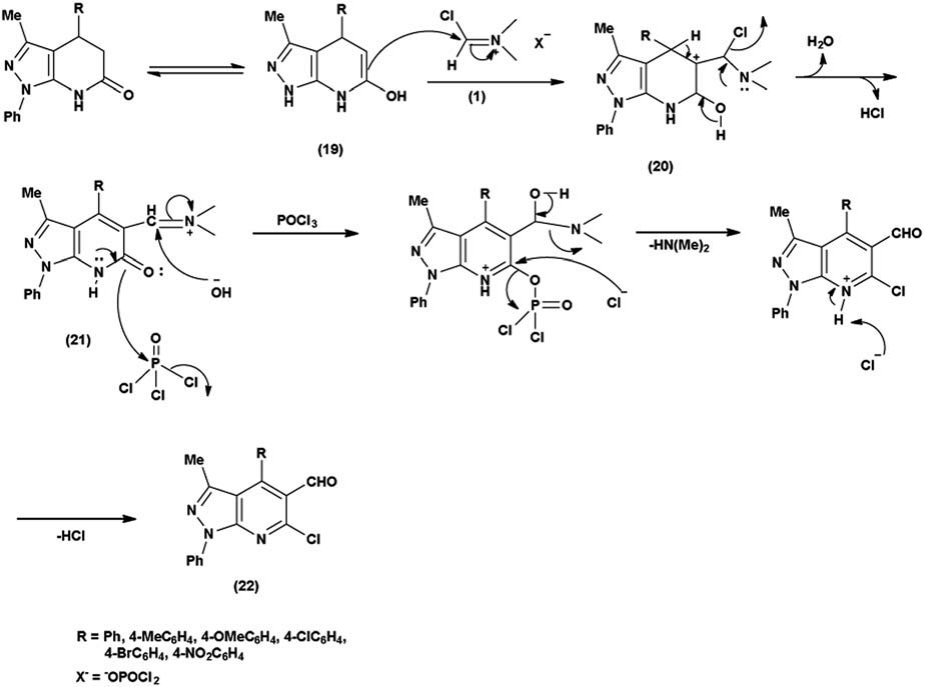
Plausible mechanism for formylation and chlorination of pyrazolo-pyridine ring.

In an initial step the V. H. reagent (DMF-POCl_3_) generated an iminium salt (1). The enol (19) of the pyrazolo-pyridine ring attack on iminium salt (1) to form an intermediate (20). The hydrolysis of the intermediate (20) afforded the formylated product (22) subsequently. Further, the intermediate (21) reacts with POCl_3_, introducing a chlorine atom in the intermediate (22). Through these orchestrated transformations, formylated as well as chlorinated pyrazolo-pyridines are achieved, showcasing the versatility of the V. H. reagent in functionalizing complex heterocyclic structures^91^ (Scheme 55).

**Scheme 56:**
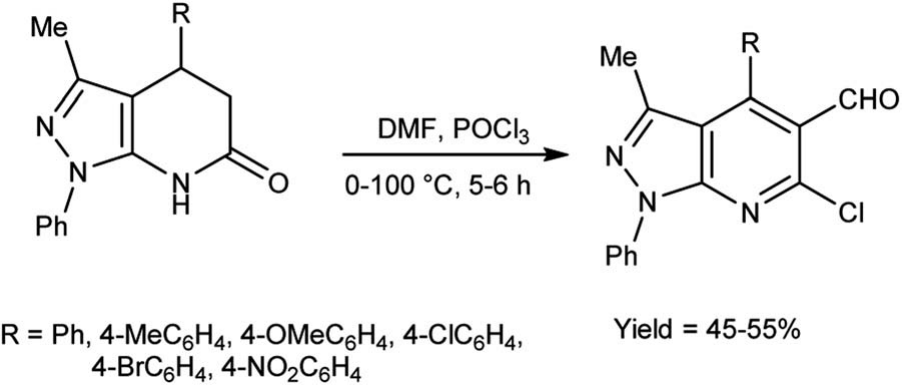
Synthesis of 6-chloropyrazolo[3,4-*b*]pyridine-5-carbaldehydes.

In 2010, Quiroga and co-workers^91^ described the synthesis of 6-chloropyrazolo[3,4-*b*]pyridine-5-carbaldehyde using V. H. reagent in ice-bath for 30 minutes followed by stirring the reaction mixture at 100 °C for 5 hours, in moderate yield. The resulting carbaldehydes have been used as precursors for the synthesis of chalcones analogue and bipyrazole[3,4-b:4^’^,3^’^-*e*]pyridines (Scheme 56).

### 4.5 Synthesis of indole carbaldehyde

**Fig. 21:**
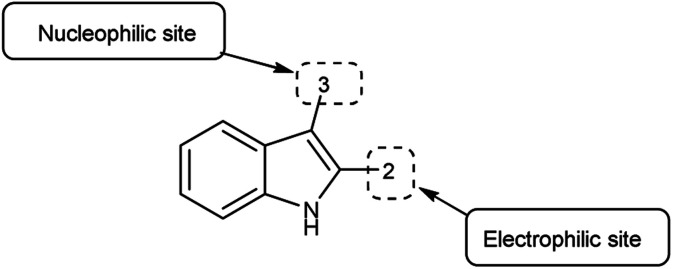
Indole.

**Fig. 22:**
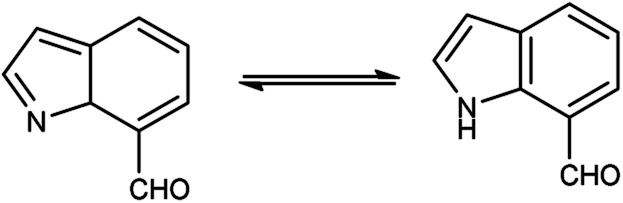
Tautomer's of indole carbaldehyde.

**Fig. 23:**
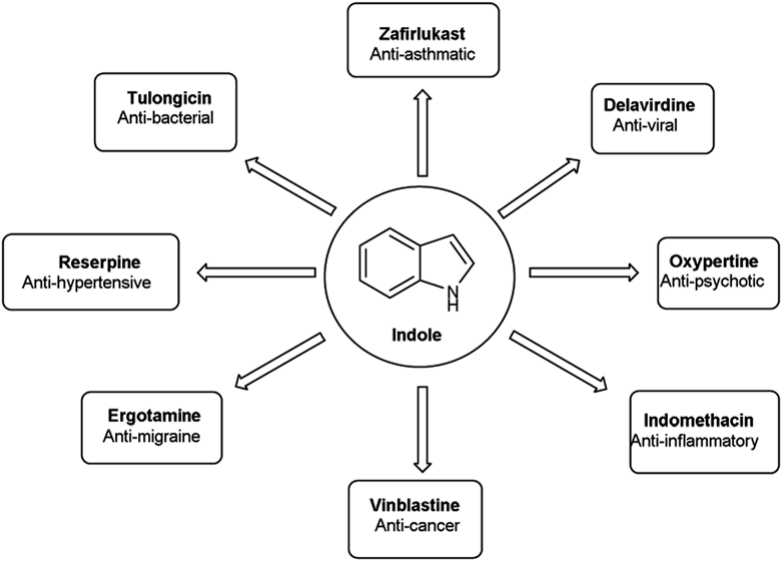
Indole based drugs available in the market.

An organic molecule with the chemical formula C_8_H_7_N is called indole containing bicyclic ring system consists of a five-membered pyrrole ring fused to a six-membered benzene ring.^92^ Because pyrrole ring in indole is more electron rich than the benzene ring, electrophile attack always occurs at five-membered ring in normal circumstances (Fig. 21). The different tautomeric forms of the ring have been demonstrated in Fig. 22. It has been used as therapeutic agents in medicinal chemistry and displayed anti-cancer, anti-oxidant, anti-rheumatoid and anti-HIV activities *etc.* (Fig. 23).

**Scheme 57:**
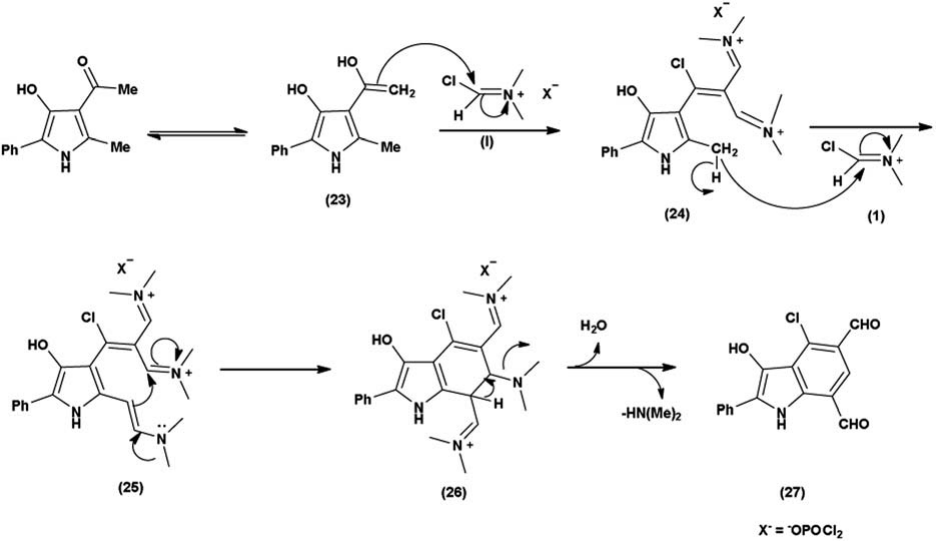
Plausible mechanism for formylation and chlorination of indole ring.

The proposed mechanism involves a series of interconnected reactions, starting from the addition of (23) to iminium salt (1), progressing through iminoalkylation and cyclization of bis-iminium salt (24) to enamine (25), followed by the elimination of dimethylamine. The resulting bis-iminium salt (26) undergoes hydrolysis, culminating in the synthesis of 2-aryl-4-chloro-3-hydroxy-1*H*-indole-5,7-dicarbaldehydes (27) as the final product^93^ (Scheme 57).

**Scheme 58:**
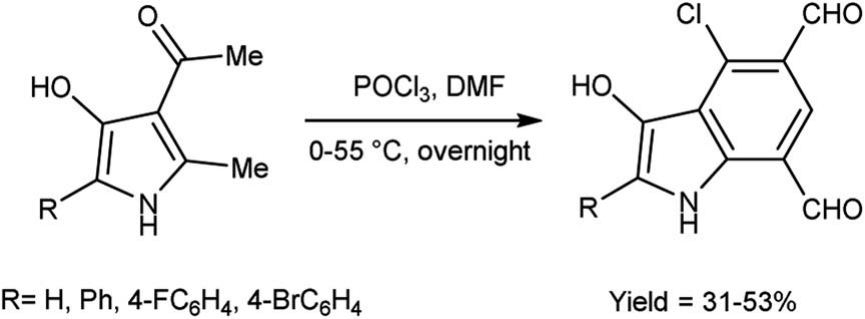
Synthesis of 2-aryl-4-chloro-3-hydroxy-1*H*-indole-5,7-dicarbaldehydes.

In 2010, Eftekhari *et al.*^93^ reported 2-aryl-4-chloro-3-hydroxy-1*H*-indole-5,7-dicarbaldehydes from 3-acetyl-5-aryl-4-hydroxy-2-methyl-1*H*-pyrroles using V. H. reagent in cold condition for 1 hour followed by stirring the reaction mixture at 55 °C for 2 hours and further stirred overnight at room temperature in average yield. The resulted products were expected to display a wide range of biological activities (Scheme 58).

**Scheme 59:**
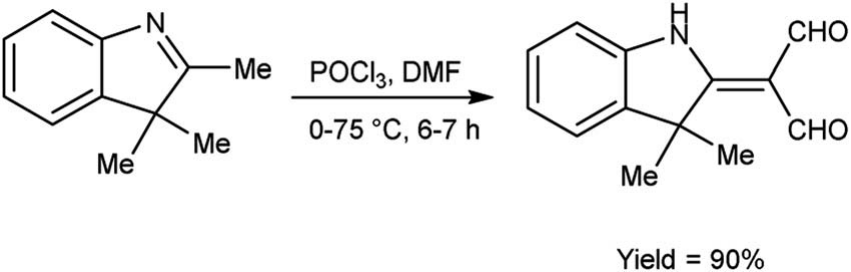
Synthesis of 2-(3,3-dimethyl-1,3-dihydro-indol-2-ylidene)-malonaldehyde.

In 2022, Faraj and group^94^ provided an efficient method for synthesis of 2-(3,3-dimethyl-1,3-dihydro-indol-2-ylidene)-malonaldehyde from 2,3,3-trimethyl-3*H*-indole using V. H. reagent (DMF-POCl_3_) in ice-bath for 1 hour followed by stirring the reaction mixture at 75 °C for 6 hours, in excellent yield. The compound 3-(2-decyloxy-phenylimino)-2-(3,3-dimethyl-1,3-dihydro-indol-2-ylidene)-propionaldehyde was synthesized using this carbaldehyde as precursor. The synthesized compounds were screened against anti-fungal and anti-bacterial activities. Amongst the synthesized compounds, 3-(2-decyloxy-phenylimino)-2-(3,3-dimethyl-1,3-dihydro-indol-2-ylidene)-propionaldehyde and 3-(2-dodecyloxy-phenylimino)-2-(3,3-dimethyl-1,3-dihydro-indol-2-ylidene)-propionaldehyde displayed good anti-fungal and anti-bacterial activity against *Fusarium oxysporum*, *B. cereus*, *Enterobacter aerogenes*. These compounds have moderate solubility in water and higher kraft temperature which enhanced the hydrophobicity of surfactants (Scheme 59).

**Scheme 60:**
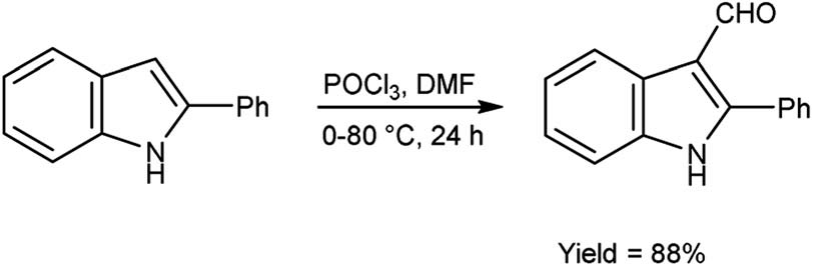
Synthesis of 2-phenyl-1*H*-indole-3-carbaldehyde.

In 2022, Husain *et al.*^95^ synthesized 2-phenyl-1*H*-indole-3-carbaldehyde from 2-phenyl-1*H*-indole using V. H. reagent (DMF-POCl_3_) in ice-bath followed by stirring the reaction mixture at 80 °C for 24 hours, in excellent yield. Further, indolyl-imidazolone hybrids were prepared by using this carbaldehyde. The resulting compounds were screened for their anti-analgesic and anti-inflammatory potential. Among the synthesized compounds, 3-(2,4-dinitrophenyl)-2-phenyl-5-[(2-phenyl-1*H*-indol-3-yl)methylene]-4*H*-imidazole-4-one and 3-(3-hydroxpropyl)-2-phenyl-5-[(2-phenyl-1*H*-indol-3-yl)methylene]-4*H*-imidazole-4-one exhibited good efficacy against inflammation and treating pain when compared to standard drug indomethacin which was further confirmed by molecular docking studies (Scheme 60).

### 4.6 Synthesis of thieno-pyridine carbaldehyde

**Fig. 24:**
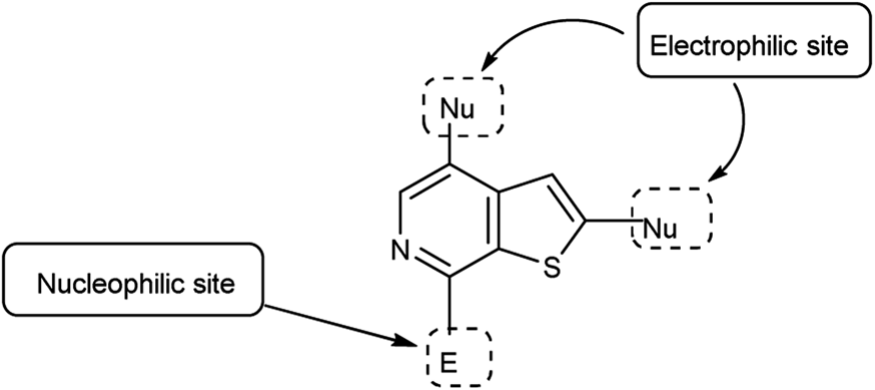
Thieno-pyridine.

**Fig. 25:**
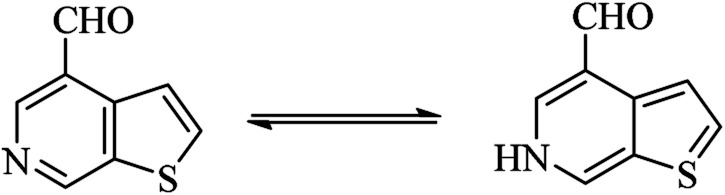
Tautomer's of thieno-pyridine carbaldehyde.

**Fig. 26:**
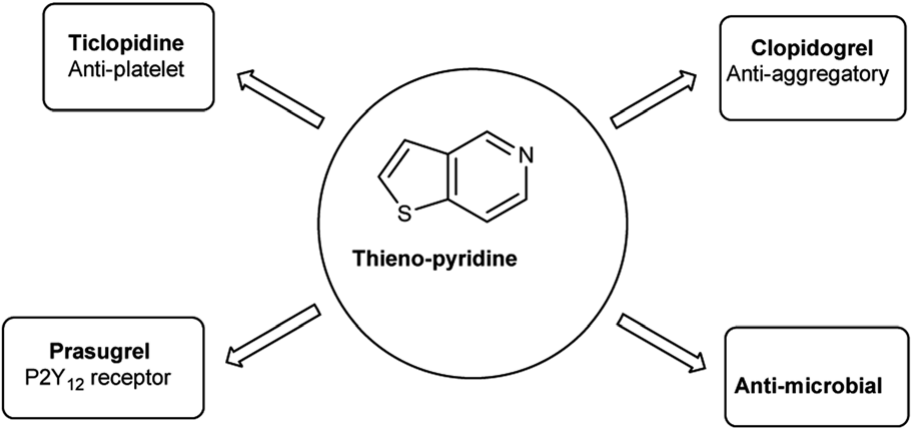
Thieno-pyridine based drugs.

Thieno-pyridine^96^ is a bicyclic compound with two fused rings, pyridine ring act as nucleophilic site whereas thiophene ring act as electrophilic site (Fig. 24). The tautomeric structures of these carbaldehyde have been shown in Fig. 25. Various drugs bearing these moieties are used to cure anxiety, depression, bacterial infection, inflammation, leishmaniasis, malaria and autoimmune disorders (Fig. 26).

**Scheme 61:**
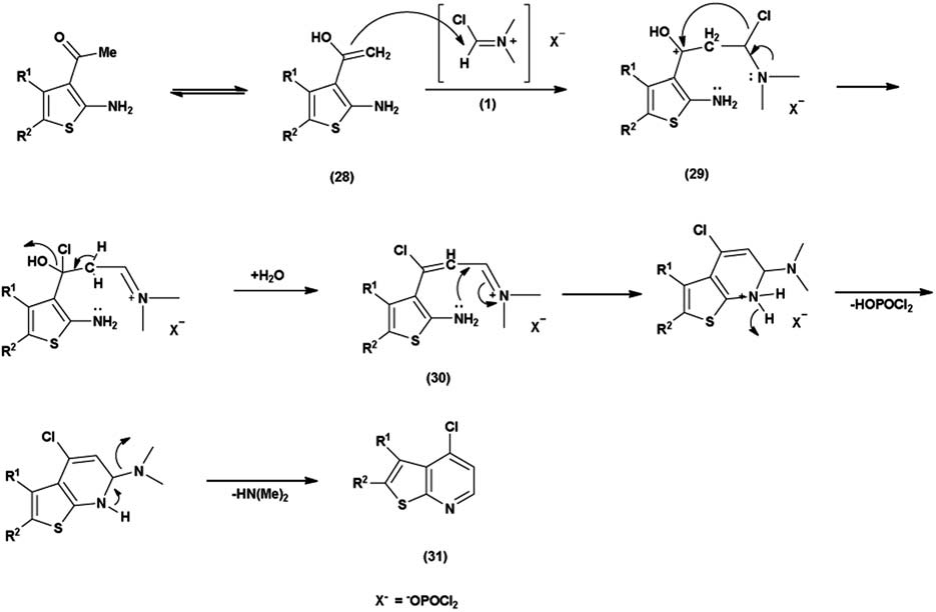
Plausible mechanism for chlorination of thieno-pyridine ring.

The chlorination of a thieno-pyridine ring with the V. H. reagent involves the activation of *N*,*N*-dimethylformamide (DMF) by phosphorus oxychloride (POCl_3_). The methyl group, depicted active enolizable proton in its mesomeric form (28). The resulting C=C bond of the enol form (28) is poised for attack V. H. reagent (1) by forming intermediate (29). The chloride ion then replaces a hydroxy group, forming a resonance stabilized intermediate (30). Further, the lone pair of the nitrogen atom attacks on the electrophilic carbon to form 6-membered ring during cyclization with the removal of dimethyl amine group for the chlorination of thieno-pyridine ring (31)^97^ (Scheme 61).

**Scheme 62:**
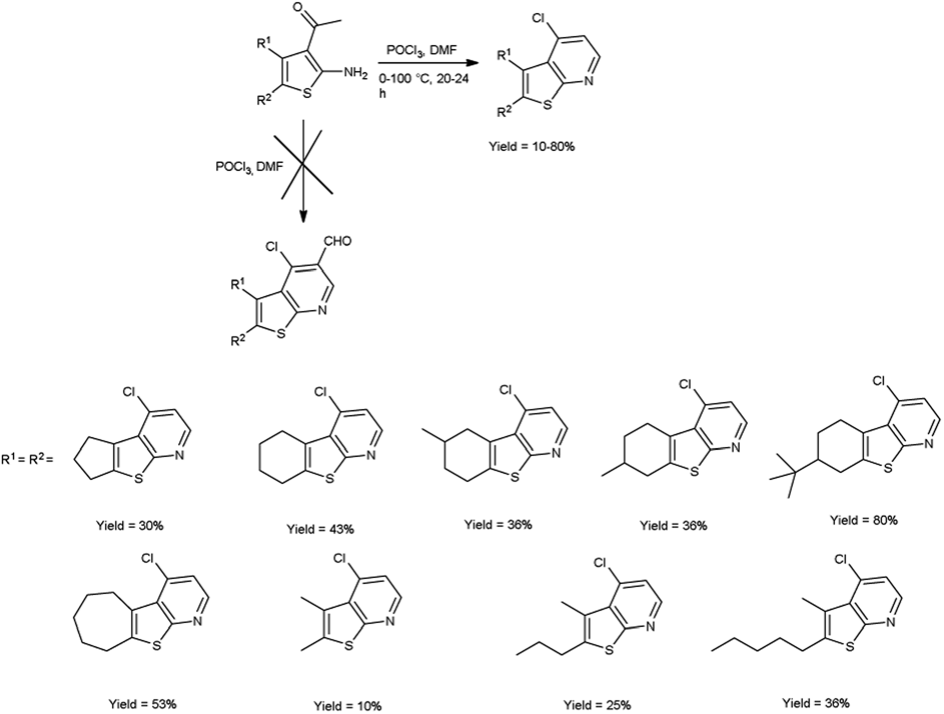
Synthesis of 4-chlorothieno[2,3-*b*]pyridines.

In 2016, Abdelwahab and group^97^ synthesized some new 4-chlorothieno[2,3-*b*]pyridine derivatives from 3-acetyl-2-aminothiophene using V. H. reagent (DMF-POCl_3_) at 0 °C for 15 minutes followed by stirring the reaction mixture at 100 °C for 20 to 24 hours, in poor to good yield. This reaction pathway enables the fabrication of innovative 4-chlorothieno[2,3-*b*]pyridine, achieved through the judicious assembly of elementary building blocks. Additionally, *N*-phenyl-5,6,7,8-tetrahydrobenzo[4,5]thieno[2,3-*b*]pyridin-4-amine were prepared by interacted with aniline *via* a C–N cross-coupling reaction using 4-chlorothieno[2,3-*b*]pyridine catalyzed by palladium to produce the coupled product (Scheme 62).

**Scheme 63:**
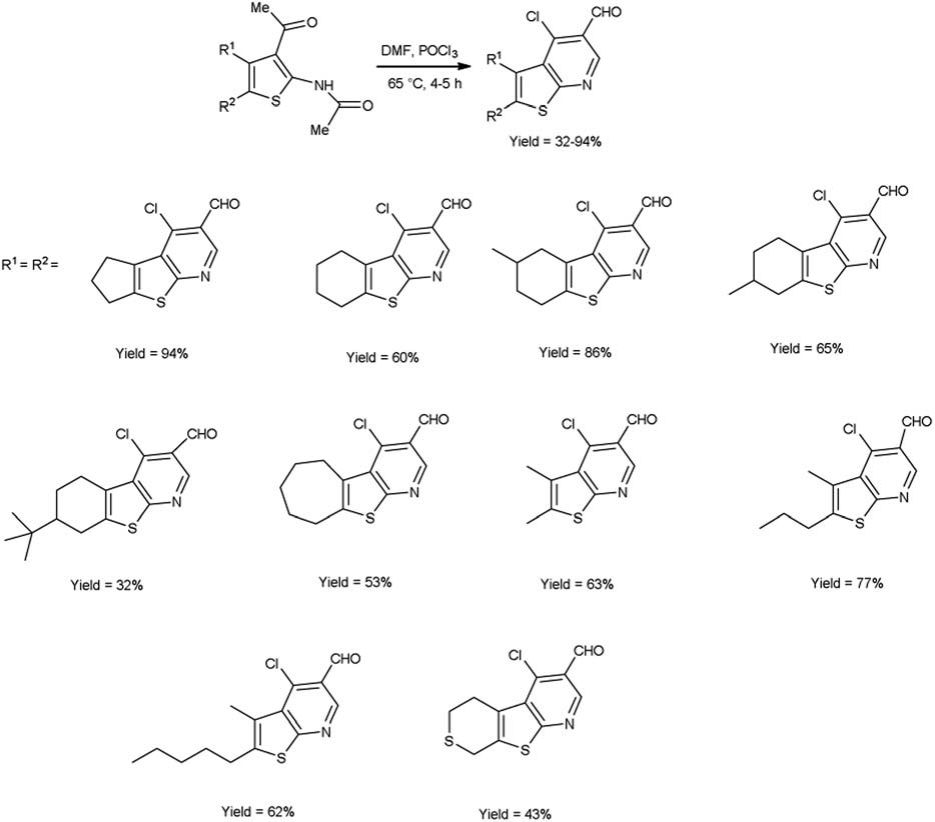
Synthesis of 4-chloro-3-formylthieno[2,3-*b*]pyridiness.

In 2017, Abdelwahab *et al.*^98^ also synthesized few 4-chloro-3-formylthieno[2,3-*b*]pyridine from *N*-protected *N*-(3-acetylthiophen-2-yl)acetamide using V. H. reagent (12 equivalent) by stirring the reaction mixture at 65 °C for 4 to 5 hours, in moderate to excellent yield. While at higher temperatures (100 °C) using six equivalent of V. H. reagent, only the formylation of *N*-(3-acetylthiophen-2-yl)acetamide was reported. Modulation of the reaction parameters led to the generation of unformylated derivatives in superior yield as compared to the reaction involving aminothiophene devoid of protective groups (Scheme 63).

### 4.7 Synthesis of miscellaneous compounds

**Scheme 64:**
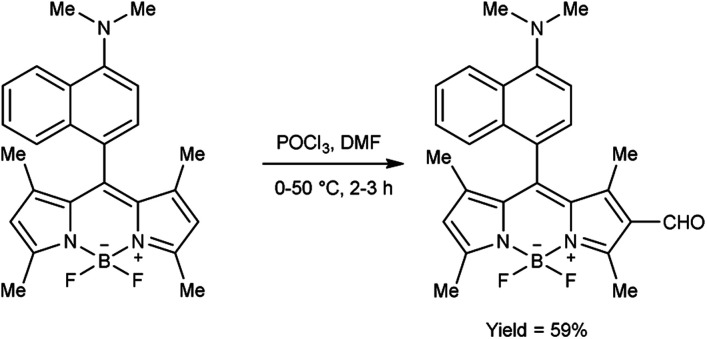
Synthesis of 3-difluoroborodipyrromethene carbaldehyde.

In 2018, Goncalves and group^99^ synthesized 3-difluoroborodipyrromethene carbaldehyde from 3-difluoroborodipyrromethene (BODIPY) using V. H. reagent at 0 °C to room temperature for 30 minutes followed by stirring the reaction mixture at 50 °C for 2 hours, in good yield. The resulting compound was further treated with *o*-phenylenediamine to form benzoimidazolyl-3-difluoroborodipyrromethene, which exhibited colorimetric chemo sensor activity when analysed in presence of several ions (AcO^−^, F^−^, Cl^−^, CN^−^, NO_3_^−^, BzO^−^, H_2_PO_4_^−^, HSO_4_^−^, Cu^2+^, Co^2+^, Pd^2+^, Ni^2+^, Ca^2+^, Hg^2+^, Zn^2+^, Fe^2+^, Fe^3+^ and Na^+^*etc.*) with environmental, biomedical and analytical relevance (Scheme 64).

**Scheme 65:**
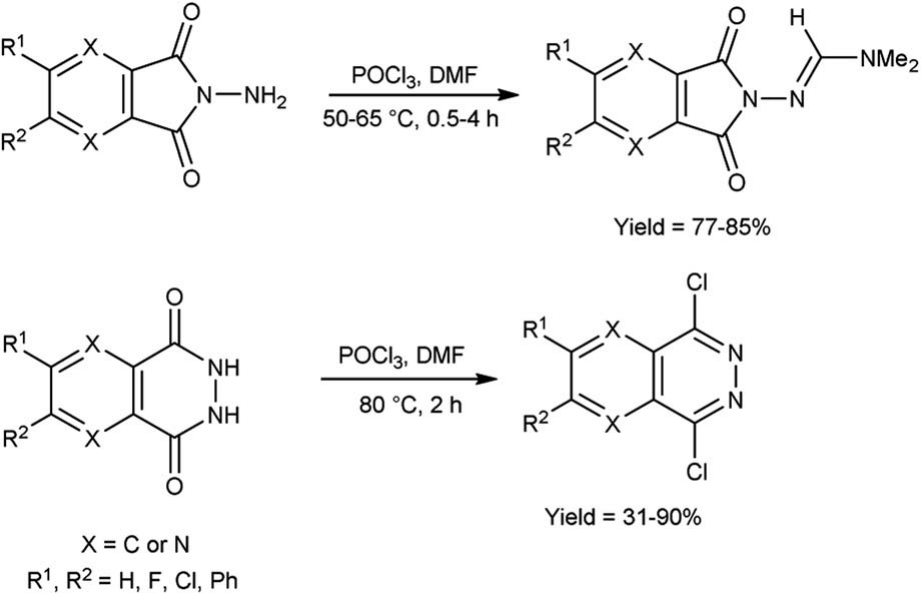
Synthesis of phthalazine-1,4-diones and *N*-aminophthalimides.

In 2021, Chung and co-workers^100^ synthesized *N*,*N*-dimethylformimidamide-7-aminopyrazolopyrrolopyridine-6,8-dione and dichloropyridazine from *N*-aminophthalimides and *N*-phthalazine-1,4-diones by using V. H. reagent by stirring the reaction mixture at 50 to 80 °C for 0.5 to 4 hours, in moderate to excellent yield. Using the V. H. reagent, the structural difference between *N*-aminophthalimides and phthalazine 1,4-diones was successfully determined (Scheme 65).

**Scheme 66:**
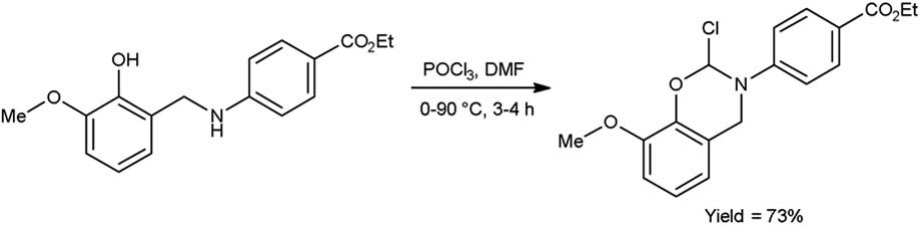
Synthesis of 4-(2-chloro-8-methoxy-2*H*-benzo[*e*][1,3]oxazin-3(4*H*)-yl)benzoate.

In 2020, Saoud *et al.*^101^ synthesized 4-(2-chloro-8-methoxy-2*H*-benzo[*e*][1,3]oxazin-3-(4*H*)-yl)benzoate from ethyl-4-((2-hydroxy-3-methoxybenzyl)amino)benzoate using V. H. reagent at 0 °C for 1 hour followed by stirring the reaction mixture at 90 °C for 3 hours, in good yield. The synthesized compound was further treated with hydrazine hydrate and benzaldehyde to form benzohydrazide. The resultant compounds were screened for their anti-microbial activity against *Acinetobacter calcoaceticus* (ATCC 23055), *E. coli* (ATCC 10538), *P. aeruginosa* (ATCC 15442), *S. typhimurium* (ATCC 14028), *B. subtilis* (ATCC 6051), *Enterococcus faecalis* (ATCC 29212), *Streptococcus pyogenes* (ATCC 19615) and *S. aureus* (ATCC 29213) using minimal inhibitory concentrations (MICs) method. Amongst the synthesized compounds, 4-(2-chloro-8-methoxy-2*H*-benzo[1,3]oxazin-3(4*H*)-yl)-*N*′-(4-nitrobenzylidene)benzohydrazide and 4-(2-chloro-2*H*-benzo[*E*][1,3]oxazin-3(4*H*)-yl)-*N*′-(3,5-dichlorobenzylidene)benzohydrazide displayed high anti-bacterial activity against both Gram-negative and Gram-positive bacteria (Scheme 66).

**Scheme 67:**
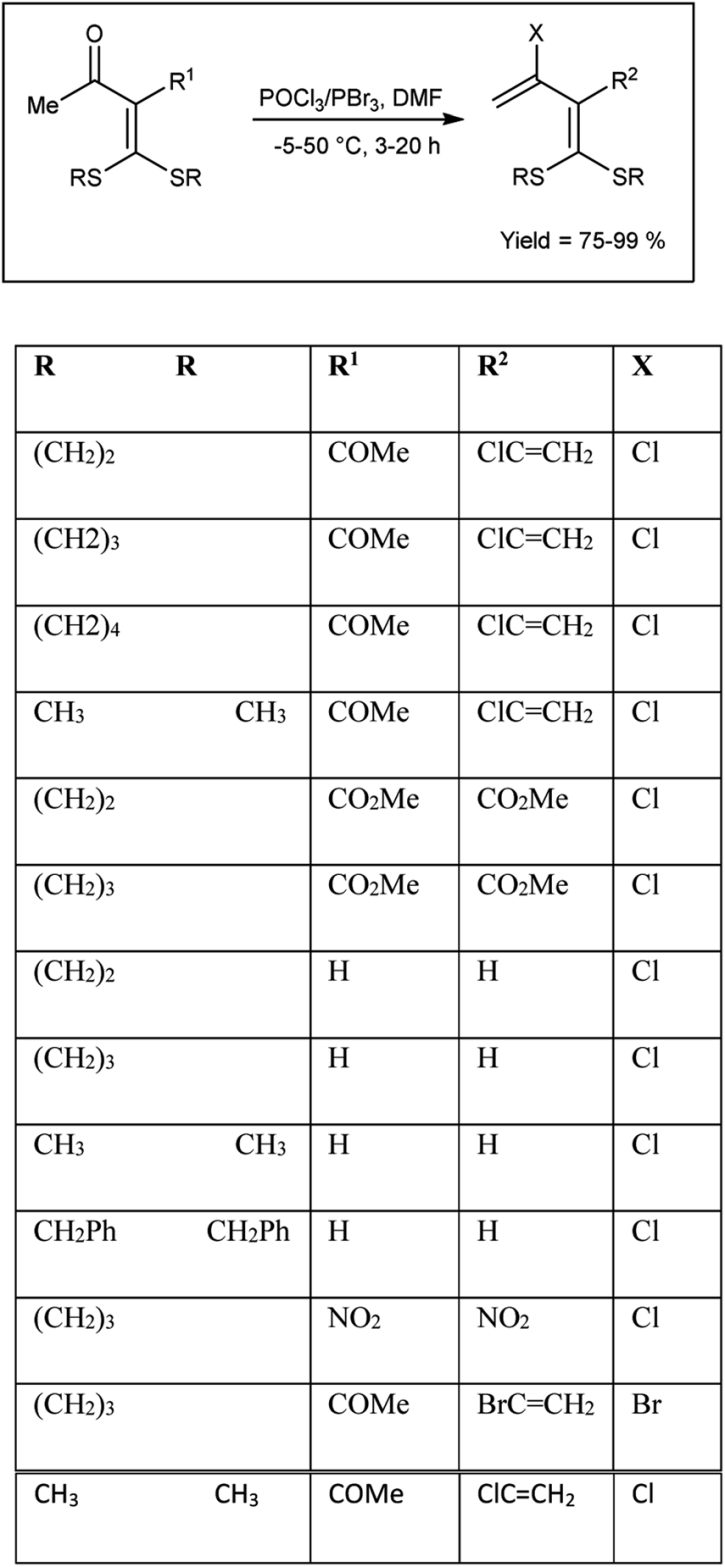
Synthesis of α-chlorovinyl/ethynyl ketene dithioacetals.

In 2005, Dong and group^102^ synthesized some α-chlorovinyl ketene dithioacetals from oxo ketene dithioacetals using V. H. reagent at −5 °C for 10 to 15 minutes followed by stirring the reaction mixture up to 50 °C for 3–20 hours, in good to excellent yield. The resulting compounds were used as precursors for the synthesis of α-ethynyl ketene dithioacetals. It is evident that electron-donating groups in proximity to the carbonyl exhibit an activating influence on carbonyl compounds, thereby facilitating the V. H. reaction. On the other hand, strong electron-withdrawing substituents at R^1^ in the substrate did not support the V. H. reaction (Scheme 67).

**Scheme 68:**
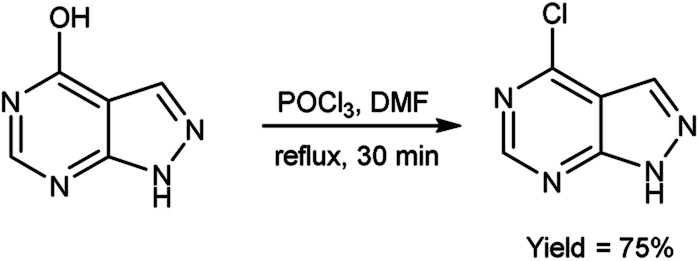
Synthesis of 4-chloro-1*H*-pyrazolo[3,4-*d*] pyrimidine.

In 2017, Chami and group^103^ synthesized 4-chloro-1*H*-pyrazolo[3,4-*d*]pyrimidine from 1*H*-pyrazolo[3,4-*d*]pyrimidin-4-one using V. H. reagent under reflux condition for 30 minutes, in good yield. The resulting compound was screened to analyzed the morphology of steel surface using corrosion monitoring methods. Potentiodynamic polarization curves were also showed potential as corrosion inhibitor of steel. The study relieved that increasing the concentration of inhibitor leads to drop in corrosion current density and an increase in inhibition efficacy (*η*_Tafel_%), implying that inhibitor molecules adsorbed at the surface of mild steel to form a protective layer (Scheme 68).

**Scheme 69:**
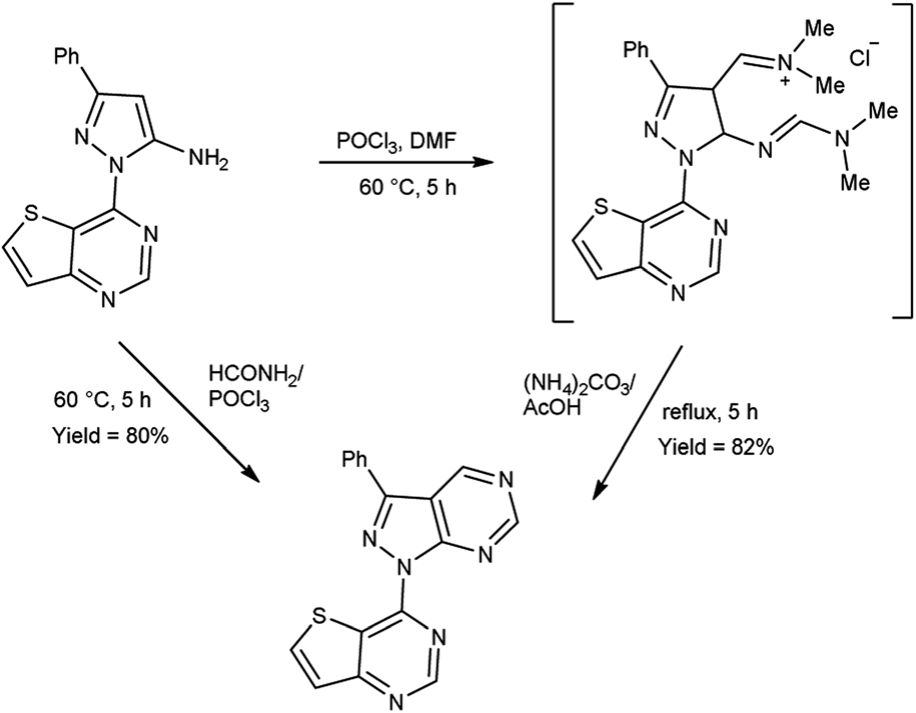
Synthesis of 4-(3-phenyl-1*H*-pyrazolo[3,4-*d*]pyrimidin-1-yl)thieno[3,2-*d*]pyrimidine.

In 2020, Noh and co-workers^104^ synthesized few 4-(3-phenyl-1*H*-pyrazolo[3,4-*d*]pyrimidin-1-yl)thieno[3,2-*d*]pyrimidine from 3-phenyl-1-(thieno[3,2-*d*]pyrimidin-4-yl)-1*H*-pyrazol-5-amine using V. H. reagent and ammonium carbonate by stirring the reaction mixture at 60 °C for 5 hours, in excellent yield. However, the resulting compound was also synthesized directly with the help of formamide (DMF) and phosphorus oxychloride (POCl_3_). The synthesized compound displayed good luciferase activity with IC_50_ value 2.55 μM (Scheme 69).

**Scheme 70:**
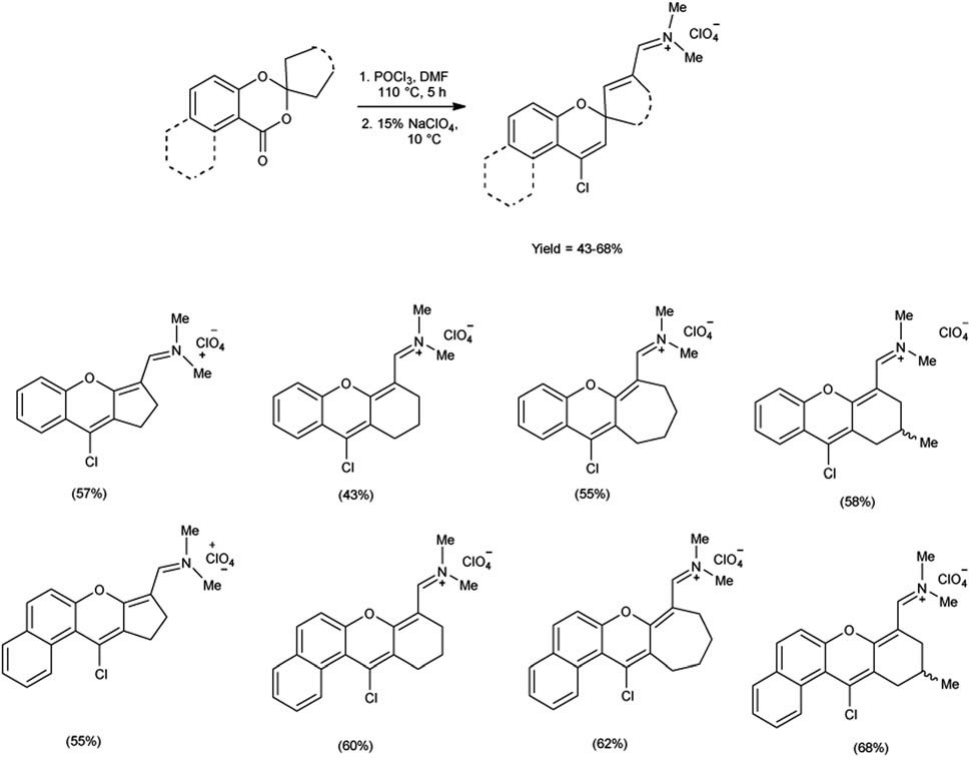
Synthesis of xanthene and benzoxanthene polyfunctional derivatives.

In 2021, Farat *et al.*^105^ synthesized some xanthene and benzoxanthene polyfunctional derivatives in form of organic perchlorates from 1,3-benzo(naphtho)dioxin-4(1)-ones using V. H. reagent (DMF-POCl_3_) by stirring the reaction mixture at 110 °C for 5 hours followed by addition of sodium perchlorate at 10 °C. The products were obtained in moderate to good yield. The observed rearrangement can be attributed to an electrophilic-induced recyclization mechanism, which is facilitated by the geminal positioning of oxygen atoms in the six-membered ring (Scheme 70).

**Scheme 71:**
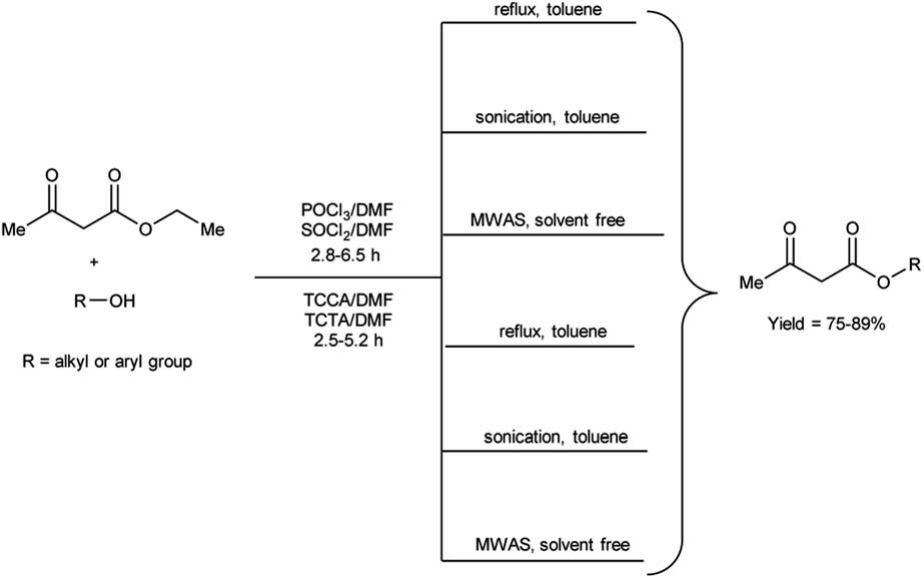
Transesterification of keto-esters.

In 2020, Duguta and group^106^ used V. H. reagent (POCl_3_/DMF or SOCl_2_/DMF) for transesterification of β-ketoesters using 1,3,5-triazine compounds like trichloroisocyanuric acid (TCCA) and triclorotriazine acid (TCTA) along with DMF (TCCA/DMF and TCTA/DMF) for 2.5 to 6.5 hours at room temperature followed by refluxing, in good to excellent yield. The V. H. reagent *i.e.*, POCl_3_/DMF or SOCl_2_/DMF was used as eco-friendly catalyst for efficient transesterification of keto-esters with various alcohols. The refined green V. H. adducts were found to be superior in contrast to classical V. H. reagent. The reaction time was further diminished and yield was enhanced when ultrasonic and microwave-assisted conditions were employed. The established methodologies facilitated expeditious access to a wide range of esters while circumventing the necessity for excessive quantities of the respective alcohols, often employed as solvents. Notably, the catalysts employed in this study, namely TCCA, TCTA and DMF are characterized by their cost-effectiveness and straightforward implementation, as they are readily available and routinely utilized as laboratory chemicals (Scheme 71).

**Scheme 72:**
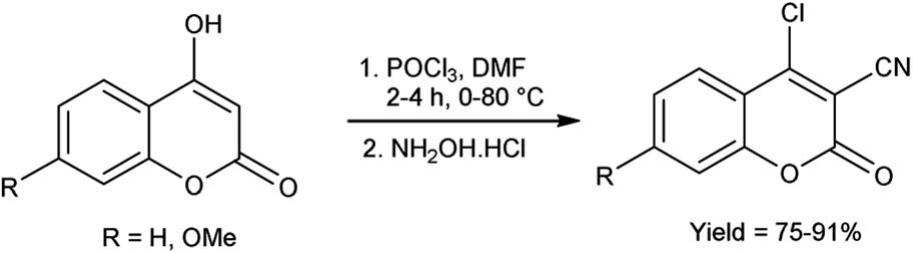
Synthesis of 6*H*, 7*H*-chromeno[3′,4′:4,5]thieno[3,2-*b*]indol-6-ones.

In 2021, Irgashev *et al.*^107^ provided the synthesis of few 4-chloro-3-cyanocoumarins from 4-hydroxycoumarins utilizing V. H. reagent and hydroxylamine hydrochloride in cold condition followed by stirring the reaction mixture at 80 °C for 2 to 4 hours, in good to excellent yield. The synthesized compound was used as excellent precursor for the synthesis of 6*H*,7*H*-chromeno[3′,4′:4,5]thieno[3,2-*b*]indol-6-ones. Additionally, coumarin derivatives were also used as precursors for the production of optoelectronics devices (Scheme 72).

**Scheme 73:**
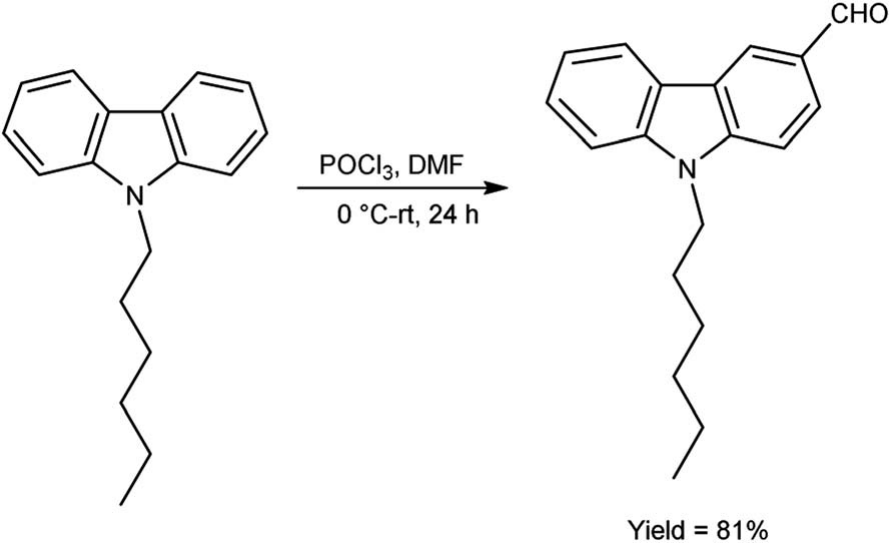
Synthesis of 9-hexyl-9*H*-carbazole-3-carbaldehyde.

In 2018, Naik and co-workers^108^ synthesized 9-hexyl-9*H*-carbazole-3-carbaldehyde from *N*-hexyl carbazole using V. H. reagent at 0 °C followed by stirring the reaction mixture at room temperature for 24 hours, in excellent yield. The resulting carbaldehyde condensed with rhodanine-3-acetic acid to form 2-((*E*)-5-((5-((*Z*)-1-cyano-2-(9-hexyl-9*H*-carbazol-3-yl) vinyl)thiophen-2-yl)methylene)-4-oxo-2-thioxothiazolidin-3-yl)acetic acid (organic dye). The synthesized dye underwent a comprehensive investigation encompassing optical, electrochemical and theoretical analyses. The photophysical characterization outcomes unveil the dyes remarkable absorption and emission spectra qualities with an observed optical band gap measuring 2.12 eV. Density functional Theory (DFT) calculations affirmed a pronounced charge separation phenomenon between the highest occupied molecular orbital (HOMO) and lowest unoccupied molecular orbital (LUMO) energy levels intrinsic to the dye molecular structure (Scheme 73).

**Scheme 74:**
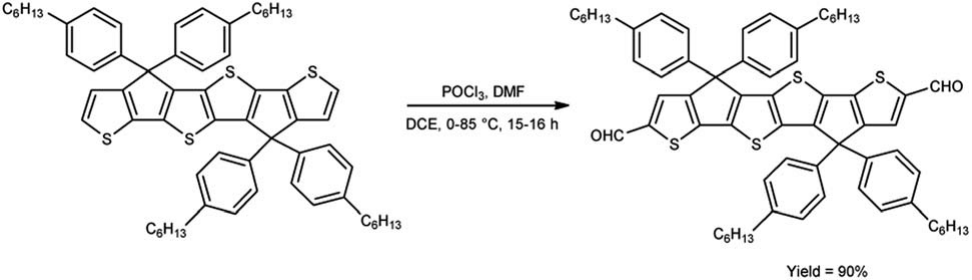
Synthesis of 3-(dicyanomethylidene)-indan-1-one.

In 2017, Gao *et al.*^109^ synthesized 3-(dicyanomethylidene)-indan-1-one using V. H. reagent at 0 °C for 30 minutes followed by stirring the reaction mixture at room temperature for 3 hours. The reaction mixture was further stirred at 85 °C for 12 hours in dichloroethane. This carbaldehyde acted as excellent precursors for the synthesis of thieno[3,2-*b*]thiophene derivatives. The resulting compound was produced in excellent yield and used in organic solar cells (OSCs) having power conversion efficiencies (PCEs) over 10% (Scheme 74).

**Scheme 75:**
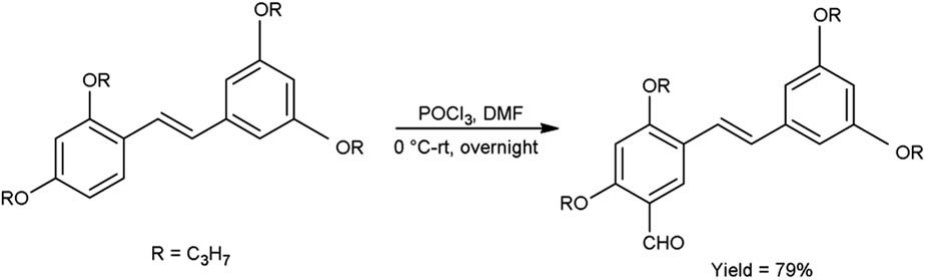
Synthesis of 2′-formyl-2,3′,4,5′-tetraisopropoxystilbene.

In 2016, Tadtong *et al.*^110^ synthesized 2′-formyl-2,3′,4,5′-tetraisopropoxystilbene from 2,3′,4,5′-tetraisopropoxystilbene using V. H. reagent at 0 °C for 2 hours in argon atmosphere. Then the reaction mixture was stirred overnight at room temperature. The resulting compounds was obtained in excellent yield. Among the synthesized compounds, 2,4-diisopropoxystilbene and 5-formyl-2,3′,4,5′-tetrahydroxystilbene (*p* < 0.05) showed high anti-oxidant activity when compared with standard drug trolox and ascorbic acid. *In vivo* study of the synthesized compound showed that compound containing electron-withdrawing functional groups exhibited good superoxide scavenging potency and stronger cell protection activity when compared to the parent compound (Scheme 75).

## 5. Conclusion

In this review, we highlighted the versatility of the Vilsmeier–Haack reagent in facilitating the formation of various five, six and fused heterocycles demonstrating its utility as a powerful tool for chemical synthesis. These synthetic approaches have not only expanded the scope of accessible heterocyclic compounds but have also enabled the rapid generation of structurally diverse molecules, offering valuable opportunities for drug discovery and development. This brief report will help researchers to explore the synthetic potential of these underutilized scaffolds and will stimulate research in this area to develop newer molecules with biological significance to be used as future drug.

## Abbreviations

V. H.: Vilsmeier–Haack
DMF: Dimethyl formamide
POCl_3_: Phosphorous trichloride
DNA: Deoxyribonucleic acid
RNA: Ribonucleic acid
MCR: Multicomponent reaction
TCCA: Trichloroisocyanuric acid
TCTA: Trichlorotriazine
NVF: *N*-Vinylformamide
*A. niger*
: *Aspergillus niger*
*E. coli*
: *Escherichia coli*
*S. aureus*
: *Staphylococcus aureus*
*B. cereus*
: *Bacillus cereus*
*P. aeruginosa*
: *Pseudomonas aeruginosa*
*C. albican*s: *Candida albicans*
*A. flavus*
: *Aspergillus flavus*
*S. typhimurium*
: *Salmonella typhimurium*

## Conflicts of interest

The authors declare no conflict of interest.

## Supplementary Material
